# Supplementary material for: Rapid two-step target capture ensures efficient CRISPR-Cas9-guided genome editing
Source: Mol Cell. Author manuscript; Available in PMC 2025 Jul 14. (PMC12258621; doi:10.1016/j.molcel.2025.03.024)
Supplement: 1 [file NIHMS2082181-supplement-1.pdf]

Supplemental information

Rapid two-step target capture ensures efficient CRISPR-Cas9-guided genome editing

Honglue Shi[1,2#], Noor Al-Sayyad[3,4#], Kevin M. Wasko[1,5#], Marena I. Trinidad[1,6], Erin E. Doherty[1,7], Kamakshi Vohra[1,7], Ron S. Boger[1,8], David Colognori[1,7], Joshua C. Cofsky[1,5,15], Petr Skopintsev[1,7], Zev Bryant[3,9*], Jennifer A. Doudna[1,2,5,7,10-14,16*]

[1]Innovative Genomics Institute, University of California, Berkeley, Berkeley, CA, USA, 94720

[2]Howard Hughes Medical Institute, University of California, Berkeley, Berkeley, CA, USA, 94720

[3]Department of Bioengineering, Stanford University, Stanford, CA, USA, 94305

[4]Department of Physics, Stanford University, Stanford, CA, USA, 94305

[5]Department of Molecular and Cell Biology, University of California, Berkeley, Berkeley, CA, USA, 94720

[6]University of California, Berkeley-University of California, San Francisco Graduate Program in Bioengineering, University of California, Berkeley, Berkeley, CA, USA, 94720

[7]California Institute for Quantitative Biosciences, University of California, Berkeley, Berkeley, CA USA, 94720

[8]Biophysics Graduate Group, University of California, Berkeley, Berkeley, CA, USA, 94720

[9]Department of Structural Biology, Stanford University Medical Center, Stanford, CA, USA, 94305

[10]Li Ka Shing Center for Genomic Engineering, University of California, Berkeley, Berkeley, CA, USA, 94720

[11]Department of Chemistry, University of California, Berkeley, Berkeley, CA, USA, 94720

[12]Molecular Biophysics and Integrated Bioimaging Division, Lawrence Berkeley National Laboratory, Berkeley, CA, USA, 94720

[13]Gladstone Institute of Data Science and Biotechnology, San Francisco, CA, USA, 94158

[14]Gladstone-UCSF Institute of Genomic Immunology, San Francisco, CA, USA, 94158

Present address:

[15]Department of Biological Chemistry and Molecular Pharmacology, Harvard Medical School, Boston, MA, USA, 02115

[16]Lead contact

[#]These authors contributed equally

*Correspondence: doudna@berkeley.edu, zevry@stanford.edu

Supplemental Figures

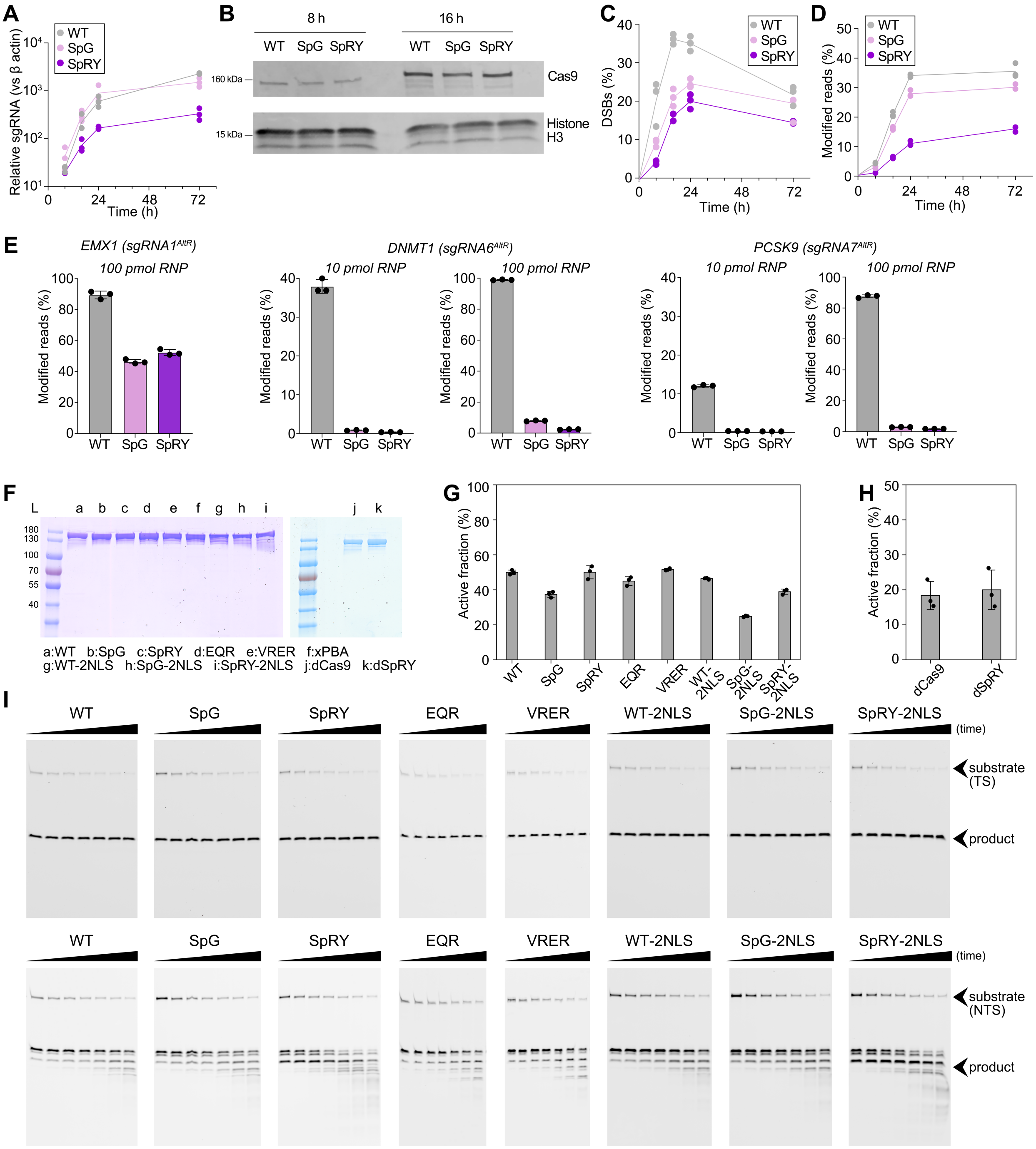

Fig. S1. Additional results from human cell experiments and quality control of Cas9 proteins (related to Fig. 1). (A) Relative quantifications of the sgRNA expression levels compared to β-actin mRNA using RT-qPCR (n=3). The >5-fold reduction of sgRNA expression at 72 h in the SpRY condition could be due to plasmid self-targeting by SpRY. **(B)** Western blot analysis of Cas9 expressions in human

cells at 8 h and 16 h post plasmid transfection. The Western blot was imaged using a Li-Cor Odyssey CLx, analyzed using Image Studio v5.2, and cropped to highlight bands of interest. **(C)** Quantifications of DNA DSB at various time points (t = 8, 16, 24, 72 h) post plasmid transfection (n=3). Note that the 8 h data is also presented as a bar graph in Fig. 1C. **(D)** Quantifications of indels at various time points (t = 8, 16, 24, 72 h) post plasmid transfection (n=3). **(E)** Quantifications of indels at 72 h post RNP nucleofection (10 or 100 pmol) targeting the *EMX1* gene, *DNMT1* gene, or *PCSK9* gene (n=3). **(F)** SDS-PAGE analysis of the Cas9 proteins used in the experiments. The left gel image was imaged using an EPSON scan and the right gel image was imaged using a BioRad ChemiDoc and both were cropped to highlight bands of interest. **(G)** Assessment of active Cas9 fractions through the percentage of cleaved dsDNA substrate (100 nM) after a 2-hour incubation with 100 nM Cas9 and 125 nM sgRNA2 at 10 mM Mg^{2+} and 37°C (n=3). **(H)** Assessment of active dCas9/dSpRY fractions based on the percentage of dsDNA substrate (100 nM) bound, measured using EMSA after 2-hour incubation with 100 nM Cas9 and 125 nM sgRNA2 at 10 mM Mg^{2+} and 37°C (n=3). **(I)** Confirmation of Cas9 proteins activity via DNA cleavage assays conducted on 15% UREA-PAGE gels, monitoring DNA cleavage over time for the (top) target strand (TS) and (bottom) non-target strand (NTS). The time points analyzed were 1 min, 2 min, 5 min, 10 min, 30 min, 60 min and 120 min. The gel images were rendered in ImageLab 6.1 (BioRad) and cropped to exclude irrelevant neighboring lanes. All error bars in **(E, G, H)** represent the standard deviation of n = 3 replicates.

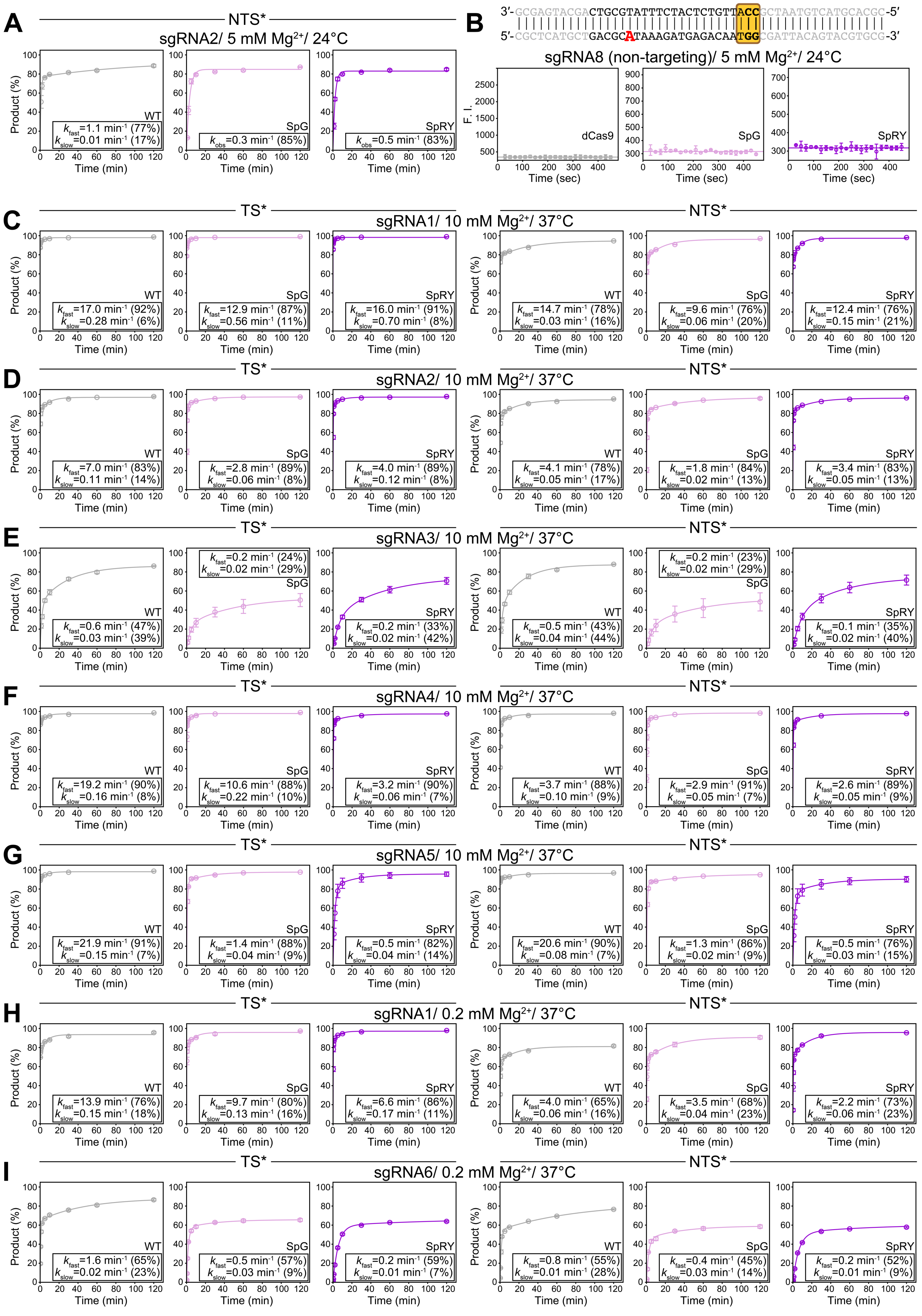

A
NTS*
sgRNA2/ 5 mM Mg2+/ 24°C
Product (%)
Time (min)
WT
kfast=1.1 min-1 (77%)
kslow=0.01 min-1 (17%)
SpG
kobs=0.3 min-1 (85%)
SpRY
kobs=0.5 min-1 (83%)
B
3′-GCGAGTACGACTGCGTATTTCTACTCTGTTACCGCTAATGTCATGCACGC-5′
5′-CGCTCATGCTGACGCATAAAGATGAGACAATGGCGATTACAGTACGTGCG-3′
sgRNA8 (non-targeting)/ 5 mM Mg2+/ 24°C
F. I.
Time (sec)
dCas9
SpG
SpRY
C
TS*
NTS*
sgRNA1/ 10 mM Mg2+/ 37°C
WT
kfast=17.0 min-1 (92%)
kslow=0.28 min-1 (6%)
SpG
kfast=12.9 min-1 (87%)
kslow=0.56 min-1 (11%)
SpRY
kfast=16.0 min-1 (91%)
kslow=0.70 min-1 (8%)
WT
kfast=14.7 min-1 (78%)
kslow=0.03 min-1 (16%)
SpG
kfast=9.6 min-1 (76%)
kslow=0.06 min-1 (20%)
SpRY
kfast=12.4 min-1 (76%)
kslow=0.15 min-1 (21%)
D
TS*
NTS*
sgRNA2/ 10 mM Mg2+/ 37°C
WT
kfast=7.0 min-1 (83%)
kslow=0.11 min-1 (14%)
SpG
kfast=2.8 min-1 (89%)
kslow=0.06 min-1 (8%)
SpRY
kfast=4.0 min-1 (89%)
kslow=0.12 min-1 (8%)
WT
kfast=4.1 min-1 (78%)
kslow=0.05 min-1 (17%)
SpG
kfast=1.8 min-1 (84%)
kslow=0.02 min-1 (13%)
SpRY
kfast=3.4 min-1 (83%)
kslow=0.05 min-1 (13%)
E
TS*
NTS*
sgRNA3/ 10 mM Mg2+/ 37°C
WT
kfast=0.6 min-1 (47%)
kslow=0.03 min-1 (39%)
SpG
kfast=0.2 min-1 (24%)
kslow=0.02 min-1 (29%)
SpRY
kfast=0.2 min-1 (33%)
kslow=0.02 min-1 (42%)
WT
kfast=0.5 min-1 (43%)
kslow=0.04 min-1 (44%)
SpG
kfast=0.2 min-1 (23%)
kslow=0.02 min-1 (29%)
SpRY
kfast=0.1 min-1 (35%)
kslow=0.02 min-1 (40%)
F
TS*
NTS*
sgRNA4/ 10 mM Mg2+/ 37°C
WT
kfast=19.2 min-1 (90%)
kslow=0.16 min-1 (8%)
SpG
kfast=10.6 min-1 (88%)
kslow=0.22 min-1 (10%)
SpRY
kfast=3.2 min-1 (90%)
kslow=0.06 min-1 (7%)
WT
kfast=3.7 min-1 (88%)
kslow=0.10 min-1 (9%)
SpG
kfast=2.9 min-1 (91%)
kslow=0.05 min-1 (7%)
SpRY
kfast=2.6 min-1 (89%)
kslow=0.05 min-1 (9%)
G
TS*
NTS*
sgRNA5/ 10 mM Mg2+/ 37°C
WT
kfast=21.9 min-1 (91%)
kslow=0.15 min-1 (7%)
SpG
kfast=1.4 min-1 (88%)
kslow=0.04 min-1 (9%)
SpRY
kfast=0.5 min-1 (82%)
kslow=0.04 min-1 (14%)
WT
kfast=20.6 min-1 (90%)
kslow=0.08 min-1 (7%)
SpG
kfast=1.3 min-1 (86%)
kslow=0.02 min-1 (9%)
SpRY
kfast=0.5 min-1 (76%)
kslow=0.03 min-1 (15%)
H
TS*
NTS*
sgRNA1/ 0.2 mM Mg2+/ 37°C
WT
kfast=13.9 min-1 (76%)
kslow=0.15 min-1 (18%)
SpG
kfast=9.7 min-1 (80%)
kslow=0.13 min-1 (16%)
SpRY
kfast=6.6 min-1 (86%)
kslow=0.17 min-1 (11%)
WT
kfast=4.0 min-1 (65%)
kslow=0.06 min-1 (16%)
SpG
kfast=3.5 min-1 (68%)
kslow=0.04 min-1 (23%)
SpRY
kfast=2.2 min-1 (73%)
kslow=0.06 min-1 (23%)
I
TS*
NTS*
sgRNA6/ 0.2 mM Mg2+/ 37°C
WT
kfast=1.6 min-1 (65%)
kslow=0.02 min-1 (23%)
SpG
kfast=0.5 min-1 (57%)
kslow=0.03 min-1 (9%)
SpRY
kfast=0.2 min-1 (59%)
kslow=0.01 min-1 (7%)
WT
kfast=0.8 min-1 (55%)
kslow=0.01 min-1 (28%)
SpG
kfast=0.4 min-1 (45%)
kslow=0.03 min-1 (14%)
SpRY
kfast=0.2 min-1 (52%)
kslow=0.01 min-1 (9%)

Fig. S2. Additional DNA cleavage assays and 2AP assays (related to Fig. 1). (A) Time-course analysis of average NTS DNA cleavage products (n=3) with sgRNA2 in 5 mM Mg^{2+} at 24°C. The average rate constants (k_{obs} for a mono-exponential decay model; k_{fast}, k_{slow} for a double-exponential decay model), with the amplitudes from the observed exponential decay are provided. For SpG and SpRY, we assume a mono-exponential decay model, as the slow phase in the double-exponential decay model is diminished and poorly defined. **(B)** (Top) The 2AP-labeled DNA construct with the 2AP residue labeled at the 15th nt from PAM in the NTS. (Bottom) Control experiments of time-course analysis of the average fluorescence signals (n=3) in the 2AP assay for sgRNA8, which is not complementary to the target dsDNA substrate, under the same conditions as Fig. 1F. These experiments were performed to determine the intercept of the exponential fitting in Fig. 1F. For WT *Spy*Cas9, catalytically dead Cas9 (dCas9) is used here. **(C-I)** Time-course analysis of average DNA cleavage products (n=3) for various guide RNA at different conditions, presented separately for TS (left) and NTS (right). The average rate constants with the amplitudes from the observed exponential decay are provided in figure legends. Note that the time-course TS cleavage analysis in Fig. S2D is also shown in Fig. 5B for direct comparison. The k_{fast} of TS cleavage in Fig. S2D, S2G and S2I are also shown in Fig. 3E as a bar graph. All error bars represent standard deviations of n = 3 replicates.

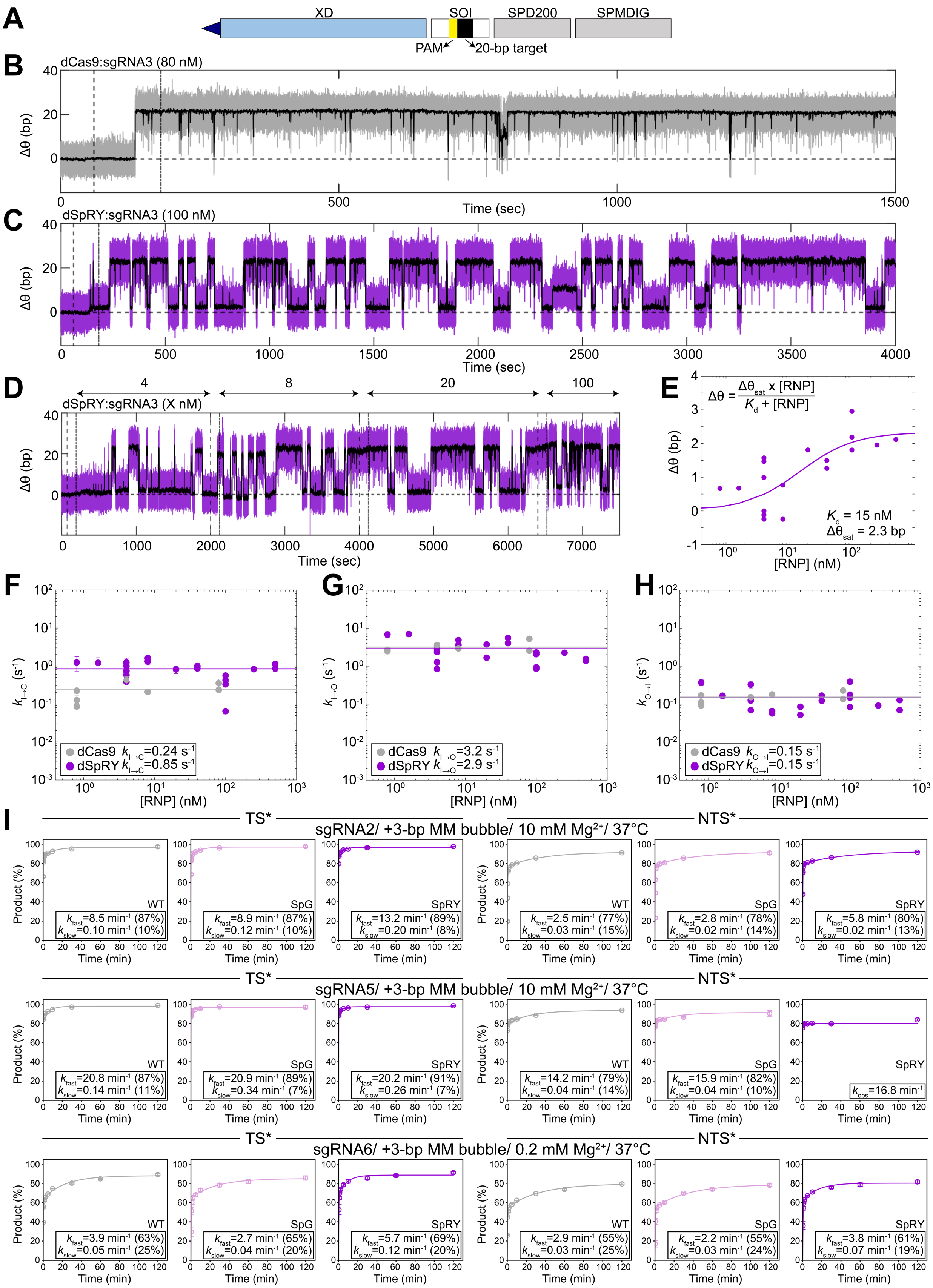
A
XD
SOI
SPD200
SPMDIG
PAM
20-bp target
B
dCas9:sgRNA3 (80 nM)
Δθ (bp)
Time (sec)
C
dSpRY:sgRNA3 (100 nM)
Δθ (bp)
Time (sec)
D
4
8
20
100
dSpRY:sgRNA3 (X nM)
Δθ (bp)
Time (sec)
E
Δθ = Δθsat x [RNP] / Kd + [RNP]
Δθ (bp)
Kd = 15 nM
Δθsat = 2.3 bp
[RNP] (nM)
F
kI→C (s-1)
dCas9 kI→C=0.24 s-1
dSpRY kI→C=0.85 s-1
[RNP] (nM)
G
kI→O (s-1)
dCas9 kI→O=3.2 s-1
dSpRY kI→O=2.9 s-1
[RNP] (nM)
H
kO→I (s-1)
dCas9 kO→I=0.15 s-1
dSpRY kO→I=0.15 s-1
[RNP] (nM)
I
TS*
NTS*
sgRNA2/ +3-bp MM bubble/ 10 mM Mg2+/ 37°C
Product (%)
Time (min)
WT kfast=8.5 min-1 (87%) kslow=0.10 min-1 (10%)
SpG kfast=8.9 min-1 (87%) kslow=0.12 min-1 (10%)
SpRY kfast=13.2 min-1 (89%) kslow=0.20 min-1 (8%)
WT kfast=2.5 min-1 (77%) kslow=0.03 min-1 (15%)
SpG kfast=2.8 min-1 (78%) kslow=0.02 min-1 (14%)
SpRY kfast=5.8 min-1 (80%) kslow=0.02 min-1 (13%)
sgRNA5/ +3-bp MM bubble/ 10 mM Mg2+/ 37°C
WT kfast=20.8 min-1 (87%) kslow=0.14 min-1 (11%)
SpG kfast=20.9 min-1 (89%) kslow=0.34 min-1 (7%)
SpRY kfast=20.2 min-1 (91%) kslow=0.26 min-1 (7%)
WT kfast=14.2 min-1 (79%) kslow=0.04 min-1 (14%)
SpG kfast=15.9 min-1 (82%) kslow=0.04 min-1 (10%)
SpRY kobs=16.8 min-1
sgRNA6/ +3-bp MM bubble/ 0.2 mM Mg2+/ 37°C
WT kfast=3.9 min-1 (63%) kslow=0.05 min-1 (25%)
SpG kfast=2.7 min-1 (65%) kslow=0.04 min-1 (20%)
SpRY kfast=5.7 min-1 (69%) kslow=0.12 min-1 (20%)
WT kfast=2.9 min-1 (55%) kslow=0.03 min-1 (25%)
SpG kfast=2.2 min-1 (55%) kslow=0.03 min-1 (24%)
SpRY kfast=3.8 min-1 (61%) kslow=0.07 min-1 (19%)

Fig. S3. Additional AuRBT analysis and DNA cleavage assays (related to Fig. 2-3). (A) DNA tethers used in AuRBT were constructed by ligating 3 PCR-generated segments (XD, SPD200, SPMDIG) and sequence of interest (SOI) from annealing ssDNA oligonucleotides. PCR primers, templates, restriction enzymes, and ssDNA oligos for making each piece are listed in Tables S15 and S16. An example of the full tether sequence is provided in Table S17. The top segment (XD) contains one 5'-Fluorescein dT (blue triangle) to make a single attachment with the magnetic bead, and two biotin-modified internal nucleotides for attachment of the rotor bead. The SPMDIG segment contains nucleotides modified with digoxigenin-dUTPs (Roche) to make multiple attachments at the coverslip. The SOI contains an NGG PAM and adjacent target sequence for Cas9 binding. SPD200 and SPMDIG contain no other NGG PAM sites. The NGG is mutated to NCG for no PAM controls. **(B-D)** Example traces from time-resolved measurements of equilibrium twist change Δθ for **(B)** 80 nM dCas9 (gray) and **(C)** 100 nM dSpRY (purple). For **(D)**, varied RNP concentrations (4, 8, 20, 100 nM dSpRY) are marked by a double-sided arrow. 250-ms averaged traces are shown in black. The vertical dashed lines (--) and dash-dot lines (-.) indicate the start and end of the flow of different concentrations of RNP into the chamber. For **(C)** and **(D)**, dSpRY:sgRNA3 induces a Δθ baseline shift. **(E)** Fit of average Δθ baseline shift and [RNP] for dSpRY:sgRNA3 binding to Target1 to a binding equation yielded an effective $K_{d,eff}$ = 15 nM. **(F-H)** Transition rate constants as a function of [RNP] with sgRNA3 on Target1 for dCas9 (gray) and dSpRY (purple) and for **(F)** $k_{I \to C}$ **(G)** $k_{I \to O}$ **(H)** $k_{O \to I}$. Solid lines depict the average value across conditions. Error bars were calculated assuming Poisson statistics. **(I)** Time-course analysis of average DNA cleavage products (n=3) for various guide RNAs on dsDNA substrates containing a 3-bp mismatch bubble adjacent to the PAM, presented separately for TS (left) and NTS (right). Error bars represent standard deviations of n = 3 replicates. The average rate constants with the amplitudes from the observed double-exponential decay are provided in figure legends. The k_{fast} of TS cleavage are also shown in Fig. 3E as a bar graph.

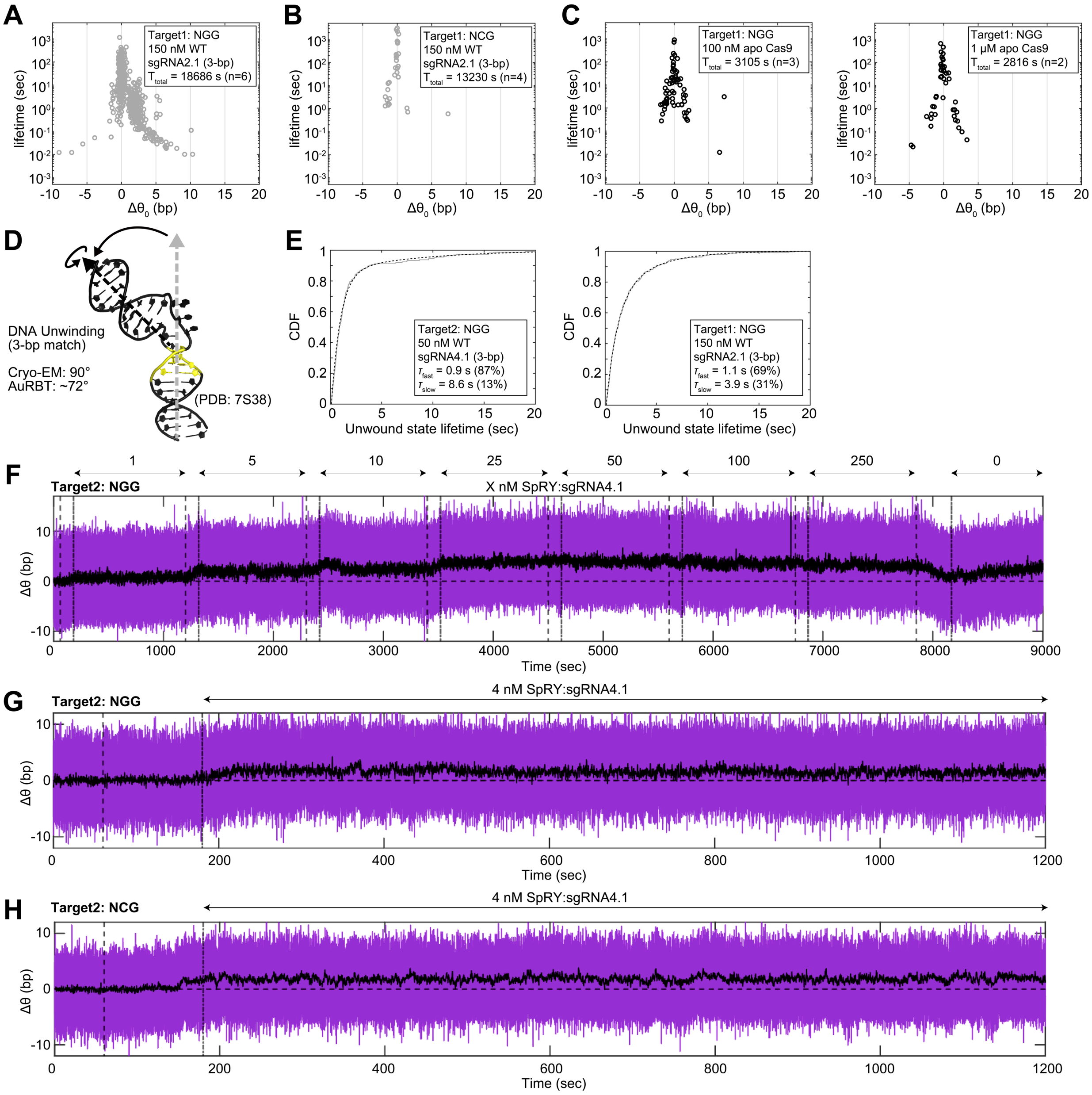

Fig. S4. Additional AuRBT analysis of early target engagement by WT *Spy*Cas9 and SpRY (related to Fig. 4). (A-C) Scatter plots illustrating the unwinding lifetime and Δθ for merged Steppi-scored states across all binding events for WT *Spy*Cas9, **(A-B)** with sgRNA2.1 containing only 3-bp match to Target1 flanking **(A)** an NGG site or **(B)** an NCG site, or **(C)** without any guide RNA. The total collection time (T_{total}) and number of DNA tethers (n) are provided in the legend. The RNP concentrations are also specified in the legend. **(D)** The degree of DNA unwinding in the Cas9 surveillance complex with 3-bp seed match (PDB: 7S38)[S1], measured by an Euler angle analysis[S2], is consistent with AuRBT results.

(E) Cumulative Distribution Function (CDF) of binding dwell times for (left) sgRNA4.1 on Target2 and (right) sgRNA2.1 on Target1. These distributions are overlaid with a 100-ms left-censored double-exponential model (dashed lines) fit using maximum likelihood estimation in MEMLET[S3]. The average lifetimes (τ) for both phases and their amplitudes are provided in the legend. These unwinding lifetimes are comparable to smFRET measurements of bound state dwell times for Cas9 with sgRNA that has 0 or 4-bp matching to the DNA next to an NGG in Singh et al.[S4], in which the τ_{fast} is 0.2-0.4 s (A_{fast}~80%) and τ_{slow} is 2-6 s (A_{slow}~20%). **(F-H)** Example traces from time-resolved measurements of equilibrium twist change Δθ for SpRY with sgRNA4.1 on the Target1 sequence flanking an **(F,G)** NGG site or an **(H)** NCG site. For **(F)** varied RNP concentrations (1, 5, 10, 25, 50, 100, 250, 0 nM dSpRY) are marked by a double-sided arrow. 250-ms averaged traces are shown in black. The vertical dashed lines (--) and dash-dot lines (-.) indicate the start and end of the flow of different concentrations of SpRY RNP into the chamber. For **(G-H)**, 4 nM RNP is used.

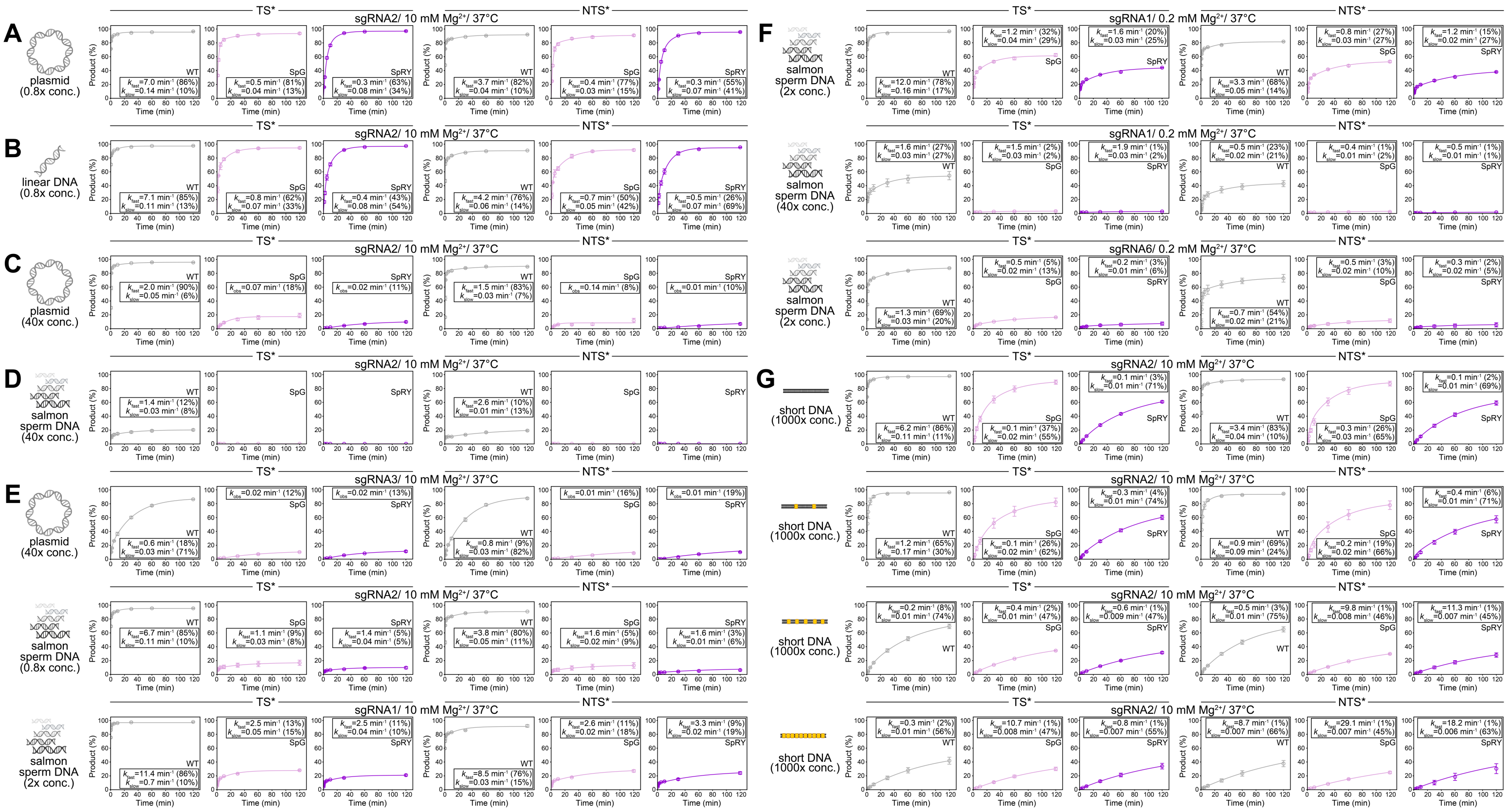

Fig. S5. DNA cleavage assays with competitors (related to Fig. 5). (A-G) Time-course analysis of average DNA cleavage products (n=3) for various guide RNAs at different conditions, presented separately for TS (left) and NTS (right). The identities and concentrations of competitors are provided on the left side of each panel. The average rate constants (k_{obs} for a mono-exponential decay model; k_{fast}, k_{slow} for a double-exponential decay model), with the amplitudes from the observed exponential decay are provided in figure legends. Note that the time-course TS cleavage analysis in Fig. S5A is also shown in Fig. 5C; the analysis in Fig. S5C is also shown in Fig. 5D and Fig. 6A; and the analysis in Fig. S5D is also shown in Fig. 5E for direct comparison. The k_{obs} for TS cleavage in Fig. S5G, assuming a mono-exponential decay, are presented as a bar graph for direct comparison in Fig. 5F. All error bars represent standard deviations of n = 3 replicates.

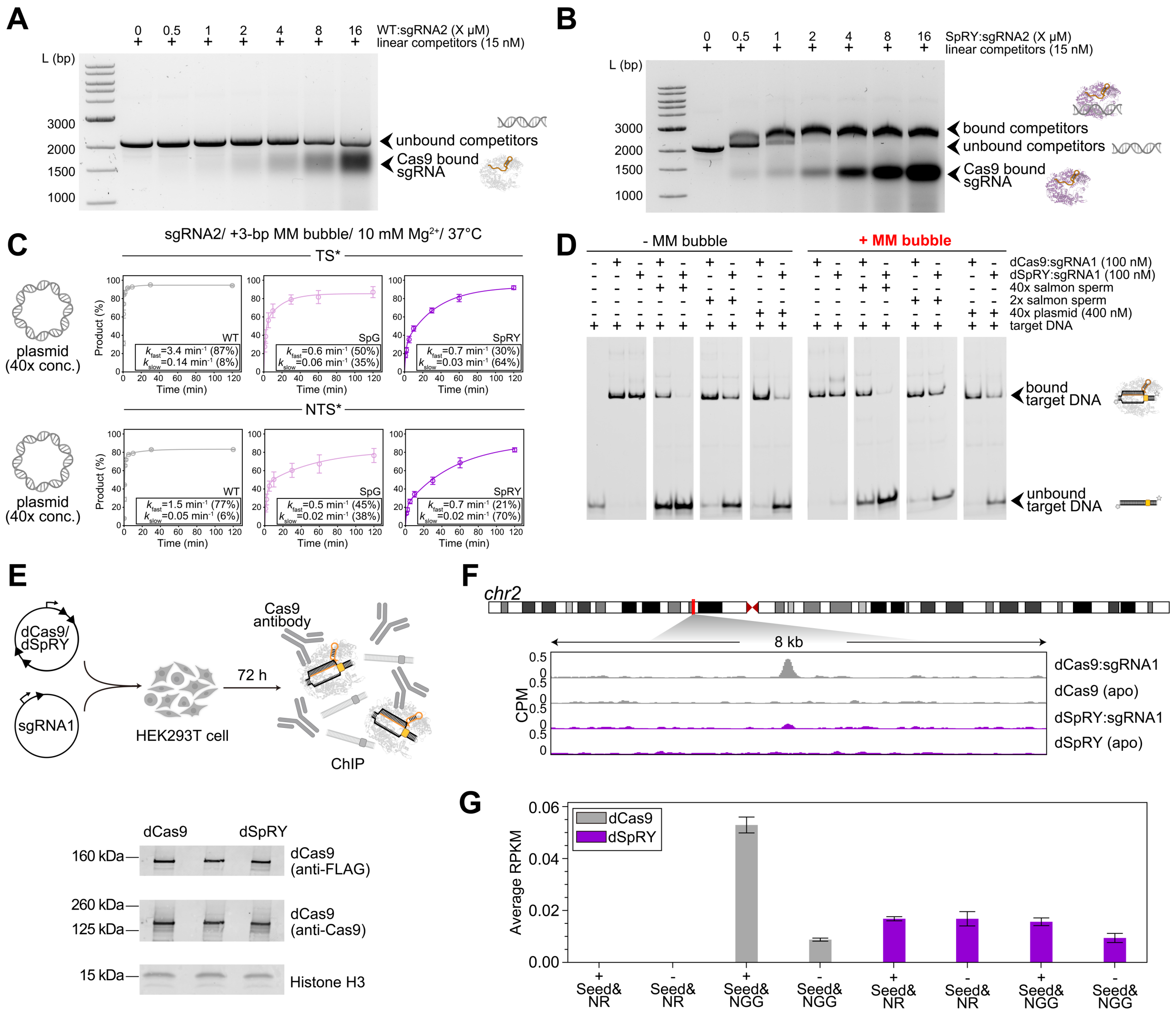

Fig. S6. Additional data for non-specific DNA interactions (related to Fig. 5-6). (A-B) EMSA analysis of WT *Spy*Cas9 and SpRY on competitor DNA without target DNA substrate. **(A)** WT *Spy*Cas9 or **(B)** SpRY with sgRNA2 are incubated with a linearized plasmid competitor DNA (2.2-kb, 15 nM) for 1 h at 10 mM Mg^{2+} and 37°C. The gel images were rendered in ImageLab 6.1 (BioRad) and cropped to exclude irrelevant neighboring lanes. **(C)** Time-course analysis of average DNA cleavage products (n=3) for sgRNA2 on dsDNA substrates containing a 3-bp mismatch bubble flanking the PAM, presented separately for TS (top) and NTS (bottom), with 40× plasmid competitors. Error bars represent standard deviations of n = 3 replicates. The average rate constants, with the amplitudes from the observed double-exponential decay are provided in figure legends. Note that the time-course TS cleavage analysis is also shown in Fig. 6A in which a mono-exponential fitting is applied for SpG and SpRY for direct comparison.

(D) Additional EMSA analysis of dCas9 and dSpRY with sgRNA1 on the target dsDNA substrate with different competitor identities and concentrations in 0.2 mM Mg^{2+} and 37°C. The gel image was rendered in ImageLab 6.1 (BioRad) and cropped to exclude irrelevant neighboring lanes. **(E)** (Top) Schematic overview of ChIP-seq experiments conducted in HEK293T cells. (Bottom) Western blot analysis showing that the anti-Cas9 antibody exhibits similar binding across different Cas9 variants. The Western blot was imaged using a Li-Cor Odyssey CLx, analyzed using Image Studio v5.2, and cropped to highlight bands of interest. **(F)** Comparison of dCas9 and dSpRY binding at the on-target site demonstrated by counts-per-million (CPM) normalized ChIP-seq data. **(G)** dCas9 exhibits more specific targeting, exhibiting higher binding to regions containing both the seed and PAM compared to those without them, as measured by ChIP-seq Reads Per Kilobase per Million mapped reads (RPKM). Average RPKM values for regions with and without the specified seed and PAM are plotted for both dCas9 and dSpRY.

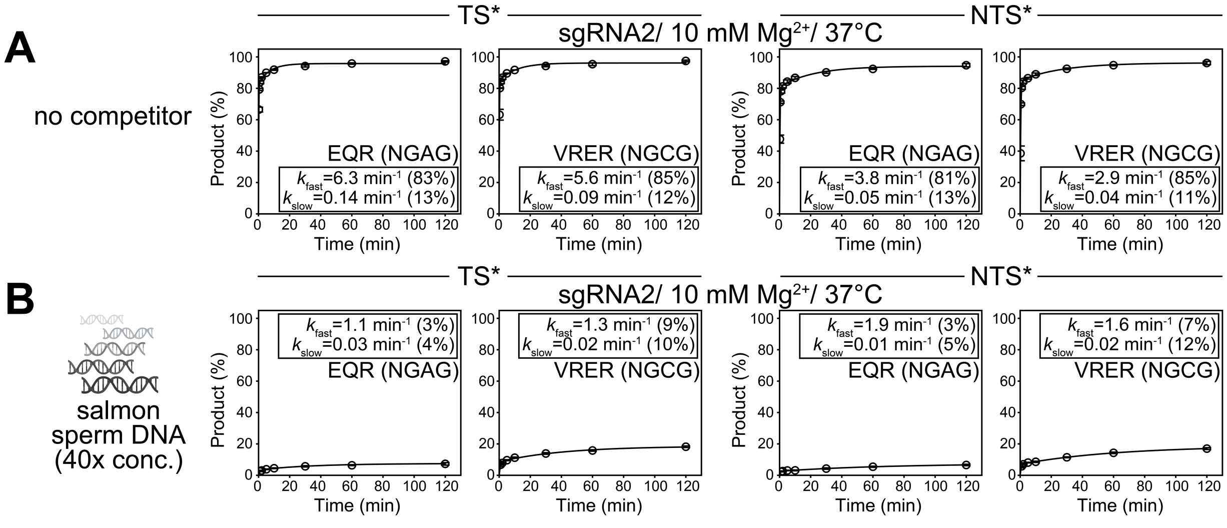

Fig. S7. DNA cleavage assays using EQR and VRER (related to Fig. 7). (A-B) Time-course analysis of average DNA cleavage products (n=3) for EQR (with NGAG PAM in the substrate) and VRER (with NGCG PAM in the substrate) with sgRNA2 at 10 mM Mg^{2+} and 37°C, presented separately for TS (left) and NTS (right), under the following conditions: **(A)** without competitor or **(B)** with salmon sperm DNA as competitor. All error bars represent the standard deviation of n = 3 replicates. The average rate constants with the amplitudes from the observed exponential decay are provided in figure legends.

Supplemental Tables

Table S1. Calculated transition rates from AuRBT experiments with 20-bp match conditions (related to Fig. 2E and Fig. 3A, B).

**Target1: dCas9 + sgRNA3 transition rate parameters**						
**Concentration [nM]**	$k_{C\to I}$ [s^{-1}]	$k_{I\to C}$ [s^{-1}]	$k_{I\to O}$ [s^{-1}]	$k_{O\to I}$ [s^{-1}]	$k_{C\to O}$ [s^{-1}]	$k_{O\to C}$ [s^{-1}]
0.8	0.131 ± 0.040	0.126 ± 0.038	2.695 ± 0.176	0.111 ± 0.007	–	–
0.8	0.046 ± 0.009	0.087 ± 0.017	2.662 ± 0.092	0.170 ± 0.006	0.002 ± 0.002	0.0002 ± 0.0002
0.8	0.052 ± 0.008	0.225 ± 0.034	2.478 ± 0.114	0.092 ± 0.004	–	–
4	0.180 ± 0.039	0.428 ± 0.093	3.604 ± 0.271	0.152 ± 0.011	–	–
8	0.408 ± 0.057	0.210 ± 0.029	2.934 ± 0.110	0.180 ± 0.007	–	–
80	4.458 ± 1.236	0.352 ± 0.098	5.228 ± 0.376	0.137 ± 0.010	–	–
80	4.156 ± 0.702	0.236 ± 0.039	2.539 ± 0.129	0.228 ± 0.012	–	–
**Average**	–	0.238 ± 0.119	3.163 ± 0.987	0.153 ± 0.045	–	–
**Target1: dSpRY + sgRNA3 transition rate parameters**						
**Concentration [nM]**	$k_{C\to I}$ [s^{-1}]	$k_{I\to C}$ [s^{-1}]	$k_{I\to O}$ [s^{-1}]	$k_{O\to I}$ [s^{-1}]	$k_{C\to O}$ [s^{-1}]	$k_{O\to C}$ [s^{-1}]
0.8	0.007 ± 0.003	1.242 ± 0.507	6.832 ± 1.189	0.371 ± 0.065	–	–
1.6	0.014 ± 0.005	1.217 ± 0.430	6.997 ± 1.032	0.167 ± 0.025	–	–
4	0.010 ± 0.002	1.002 ± 0.183	2.472 ± 0.287	0.129 ± 0.015	–	–
4	0.017 ± 0.002	0.382 ± 0.040	0.839 ± 0.060	0.069 ± 0.005	–	–
4	0.006 ± 0.001	0.728 ± 0.121	2.346 ± 0.218	0.124 ± 0.011	0.0002 ± 0.0002	0.001 ± 0.001
4	0.006 ± 0.002	0.752 ± 0.182	2.700 ± 0.346	0.129 ± 0.017	–	–
4	0.010 ± 0.003	1.238 ± 0.373	3.152 ± 0.596	0.327 ± 0.061	–	–
4	0.018 ± 0.004	0.579 ± 0.123	1.262 ± 0.182	0.151 ± 0.022	–	–
8	0.039 ± 0.008	1.596 ± 0.313	3.623 ± 0.472	0.066 ± 0.009	0.0015 ± 0.0015	0.001 ± 0.001

8	0.017 ± 0.003	1.323 ± 0.250	4.867 ± 0.480	0.057 ± 0.006	–	0.0006 ± 0.0006
20	0.026 ± 0.006	0.813 ± 0.177	3.717 ± 0.379	0.085 ± 0.009	–	–
20	0.028 ± 0.004	0.815 ± 0.118	1.665 ± 0.168	0.052 ± 0.005	0.0006 ± 0.0006	0.0005 ± 0.0005
40	0.049 ± 0.007	0.862 ± 0.121	5.511 ± 0.305	0.122 ± 0.007	0.0010 ± 0.0010	0.0004 ± 0.0004
40	0.045 ± 0.007	0.996 ± 0.164	4.037 ± 0.330	0.168 ± 0.014	–	–
100	0.101 ± 0.025	0.423 ± 0.103	2.289 ± 0.239	0.178 ± 0.019	–	–
100	0.052 ± 0.013	0.560 ± 0.140	2.064 ± 0.269	0.149 ± 0.019	–	–
100	0.056 ± 0.007	0.330 ± 0.039	0.934 ± 0.066	0.084 ± 0.006	–	–
100	0.070 ± 0.011	0.065 ± 0.010	0.853 ± 0.036	0.390 ± 0.016	0.0017 ± 0.0017	–
250	0.022 ± 0.003	0.825 ± 0.124	2.250 ± 0.205	0.092 ± 0.008	–	0.0008 ± 0.0008
500	0.051 ± 0.008	0.866 ± 0.139	1.377 ± 0.175	0.127 ± 0.016	–	–
500	0.033 ± 0.005	1.140 ± 0.166	1.552 ± 0.194	0.070 ± 0.009	–	–
**Average**	–	0.845 ± 0.376	2.921 ± 1.846	0.148 ± 0.098	–	–

Errors are calculated assuming Poisson statistics. Averages are unweighted, and errors are standard deviations.

Table S2. Fits and fit parameters for $k_{C\rightarrow I}$ versus RNP concentration (related to Fig. 3A, B).

	Fit	Fit Parameters
**dCas9**	$f([dCas9]) = k_{eff} * [dCas9]$	$k_{eff} = 0.054\ (0.052, 0.056)$ nM^{-1}s^{-1}
**dSpRY**	$f([dSpRY]) = \frac{k_{max} * [dSpRY]}{K_{d,init} + [dSpRY]}$	$K_{d,init} = 11.14\ (-1.057, 23.34)$ nM<br>$k_{max} = 0.05681\ (0.04121, 0.0724)$ s^{-1}

(95% confidence bounds)

Table S3. The energy values used in the free energy landscapes (related to Fig. 3C).

	State	Energy [k_BT]
**dCas9:sgRNA3 on Target1**	C_{free}	0
	$(C_{free}\leftrightarrow C_{bound})^{\ddagger}$	4.70
	C_{bound}	2.30
	$(C_{bound}\leftrightarrow I)^{\ddagger}$	5.39
	I	-3.04
	$(I\leftrightarrow O)^{\ddagger}$	2.80
	O	-6.07
**dSpRY:sgRNA3 on Target1**	C_{free}	0
	$(C_{free}\leftrightarrow C_{bound})^{\ddagger}$	4.70
	C_{bound}	-2.30
	$(C_{bound}\leftrightarrow I)^{\ddagger}$	7.57
	I	0.40
	$(I\leftrightarrow O)^{\ddagger}$	6.33
	O	-2.59

Assuming [RNP] = 100 nM, $k_{on,init}$(dCas9) = $k_{on,init}$(dSpRY) = 0.1 $nM^{-1}s^{-1}$, $k_{off,init}$(dCas9) = 100 s^{-1}, and the parameters shown in Fig. 3B and Tables S1 and S2, with the arbitrary shift C=7 k_BT applied to barrier heights for display.

Table S4. Plasmid vectors used in this study (related to STAR Methods).

Internal ID	System	Purpose
pKMW121	Human cells	Mammalian expression of WT *Spy*Cas9 with Golden Gate

		compatible sgRNA spacer destination vector
pKMW122	Human cells	Mammalian expression of SpG with Golden Gate compatible sgRNA spacer destination vector
pKMW123	Human cells	Mammalian expression of SpRY with Golden Gate compatible sgRNA spacer destination vector
pKMW129	Human cells	Mammalian expression of WT *Spy*Cas9 with *EMX1*-targeting sgRNA
pKMW130	Human cells	Mammalian expression of SpG with *EMX1*-targeting sgRNA
pKMW131	Human cells	Mammalian expression of SpRY with *EMX1*-targeting sgRNA
pKMW124	Human cells	Mammalian expression of dCas9
pKMW281	Human cells	Mammalian expression of dSpRY
pKMW493	Human cells	Mammalian expression of *Spy*Cas9 sgRNA, Golden Gate compatible destination vector for spacer
pKMW306	Human cells	Mammalian expression of *EMX1*-targeting sgRNA
pHS1	Bacteria	Bacterial expression of WT *Spy*Cas9
pHS23	Bacteria	Bacterial expression of SpG
pHS24	Bacteria	Bacterial expression of SpRY
pHS25	Bacteria	Bacterial expression of EQR
pHS26	Bacteria	Bacterial expression of VRER
pHS16	Bacteria	Bacterial expression of xPBA
pHS9	Bacteria	Bacterial expression of dCas9
pHS484	Bacteria	Bacterial expression of dSpRY
pHS71	Bacteria	Bacterial expression of WT *Spy*Cas9-2NLS
pHS72	Bacteria	Bacterial expression of SpG-2NLS
pHS73	Bacteria	Bacterial expression of SpRY-2NLS
pGGAselect	Bacteria	Plasmid competitor DNA used in this study.

Table S5. U6 promoter and sgRNA sequences used in human cell expression vectors (related to STAR Methods).

	Sequence (5′-3′)
U6 promoter	GAGGGCCTATTTCCCATGATTCCTTCATATTTGCATATACGAT<br>ACAAGGCTGTTAGAGAGATAATTGGAATTAATTTGACTGTAAA<br>CACAAAGATATTAGTACAAAATACGTGACGTAGAAAGTAATAA<br>TTTCTTGGGTAGTTTGCAGTTTTAAAATTATGTTTTAAAATGGA<br>CTATCATATGCTTACCGTAACTTGAAAGTATTTCGATTTCTTG<br>GCTTTATATATCTTGTGGAAAGGACGAAACACC
sgRNA (*EMX1* spacer)	**GAGTCCGAGCAGAAGAAGAA**GTTTTAGAGCTAGAAATAGCA<br>AGTTAAAATAAGGCTAGTCCGTTATCAACTTGAAAAAGTGGCA<br>CCGAGTCGGTGCTTTTTT

Bolded region represents the 20-bp sgRNA spacer; yellow highlighted region represents the sgRNA scaffold sequence

Table S6. WT *Spy*Cas9 protein sequences in different expression vectors (related to STAR Methods).

	Protein sequences
WT *Spy*Cas9 in human cell vector	MDYKDHDGDYKDHDIDYKDDDDKMAPKKKRKVGIHGVPAADKK<br>YSIGLDIGTNSVGWAVITDEYKVPSKKFKVLGNTDRHSIKKNLIGA<br>LLFDSGETAEATRLKRTARRRYTRRKNRICYLQEIFSNEMAKVD<br>DSFFHRLEESFLVEEDKKHERHPIFGNIVDEVAYHEKYPTIYHLR<br>KKLVDSTDKADLRLIYLALAHMIKFRGHFLIEGDLNPDNSDVDKL<br>FIQLVQTYNQLFEENPINASGVDAKAILSARLSKSRRLENLIAQLP<br>GEKKNGLFGNLIALSLGLTPNFKSNFDLAEDAKLQLSKDTYDDDL<br>DNLLAQIGDQYADLFLAAKNLSDAILLSDILRVNTEITKAPLSASMI<br>KRYDEHHQDLTLLKALVRQQLPEKYKEIFFDQSKNGYAGYIDGG<br>ASQEEFYKFIKPILEKMDGTEELLVKLNREDLLRKQRTFDNGSIP<br>HQIHLGELHAILRRQEDFYPFLKDNREKIEKILTFRIPYYVGPLAR<br>GNSRFAWMTRKSEETITPWNFEEVVDKGASAQSFIERMTNFDK<br>NLPNEKVLPKHSLLYEYFTVYNELTKVKYVTEGMRKPAFLSGEQ<br>KKAIVDLLFKTNRKVTVKQLKEDYFKKIECFDSVEISGVEDRFNA<br>SLGTYHDLLKIIKDKDFLDNEENEDILEDIVLTLTLFEDREMIEERL<br>KTYAHLFDDKVMKQLKRRRYTGWGRLSRKLINGIRDKQSGKTIL<br>DFLKSDGFANRNFMQLIHDDSLTFKEDIQKAQVSGQGDSLHEHI<br>ANLAGSPAIKKGILQTVKVVDELVKVMGRHKPENIVIEMARENQT<br>TQKGQKNSRERMKRIEEGIKELGSQILKEHPVENTQLQNEKLYL<br>YYLQNGRDMYVDQELDINRLSDYDVDHIVPQSFLKDDSIDNKVL<br>TRSDKNRGKSDNVPSEEVVKKMKNYWRQLLNAKLITQRKFDNL<br>TKAERGGLSELDKAGFIKRQLVETRQITKHVAQILDSRMNTKYDE<br>NDKLIREVKVITLKSKLVSDFRKDFQFYKVREINNYHHAHDAYLN<br>AVVGTALIKKYPKLESEFVYGDYKVYDVRKMIAKSEQEIGKATAK<br>YFFYSNIMNFFKTEITLANGEIRKRPLIETNGETGEIVWDKGRDFA

	TVRKVLSMPQVNIVKKTEVQTGGFSKESILPKRNSDKLIARKKD<br>WDPKKYGGFDSPTVAYSVLVVAKVEKGKSKKLKSVKELLGITIM<br>ERSSFEKNPIDFLEAKGYKEVKKDLIIKLPKYSLFELENGRKRMLA<br>SAGELQKGNELALPSKYVNFLYLASHYEKLKGSPEDNEQKQLFV<br>EQHKHYLDEIIEQISEFSKRVILADANLDKVLSAYNKHRDKPIREQ<br>AENIIHLFTLTNLGAPAAFKYFDTTIDRKRYTSTKEVLDATLIHQSI<br>TGLYETRIDLSQLGGDKRPAATKKAGQAKKKK
WT *Spy*Cas9 in bacterial expression vector	MKSSHHHHHHHHHHGSSMKIEEGKLVIWINGDKGYNGLAEVGK<br>KFEKDTGIKVTVEHPDKLEEKFPQVAATGDGPDIIFWAHDRFGG<br>YAQSGLLAEITPDKAFQDKLYPFTWDAVRYNGKLIAYPIAVEALS<br>LIYNKDLLPNPPKTWEEIPALDKELKAKGKSALMFNLQEPYFTWP<br>LIAADGGYAFKYENGKYDIKDVGVDNAGAKAGLTFLVDLIKNKH<br>MNADTDYSIAEAAFNKGETAMTINGPWAWSNIDTSKVNYGVTVL<br>PTFKGQPSKPFVGVLSAGINAASPNKELAKEFLENYLLTDEGLEA<br>VNKDKPLGAVALKSYEEELAKDPRIAATMENAQKGEIMPNIPQM<br>SAFWYAVRTAVINAASGRQTVDEALKDAQTNSSSNNNNNNNNN<br>NLGIEENLYFQSNAMDKKYSIGLDIGTNSVGWAVITDEYKVPSKK<br>FKVLGNTDRHSIKKNLIGALLFDSGETAEATRLKRTARRRYTRRK<br>NRICYLQEIFSNEMAKVDDSFFHRLEESFLVEEDKKHERHPIFGN<br>IVDEVAYHEKYPTIYHLRKKLVDSTDKADLRLIYLALAHMIKFRGH<br>FLIEGDLNPDNSDVDKLFIQLVQTYNQLFEENPINASGVDAKAILS<br>ARLSKSRRLENLIAQLPGEKKNGLFGNLIALSLGLTPNFKSNFDL<br>AEDAKLQLSKDTYDDDLDNLLAQIGDQYADLFLAAKNLSDAILLS<br>DILRVNTEITKAPLSASMIKRYDEHHQDLTLLKALVRQQLPEKYK<br>EIFFDQSKNGYAGYIDGGASQEEFYKFIKPILEKMDGTEELLVKL<br>NREDLLRKQRTFDNGSIPHQIHLGELHAILRRQEDFYPFLKDNRE<br>KIEKILTFRIPYYVGPLARGNSRFAWMTRKSEETITPWNFEEVVD<br>KGASAQSFIERMTNFDKNLPNEKVLPKHSLLYEYFTVYNELTKV<br>KYVTEGMRKPAFLSGEQKKAIVDLLFKTNRKVTVKQLKEDYFKKI<br>ECFDSVEISGVEDRFNASLGTYHDLLKIIKDKDFLDNEENEDILED<br>IVLTLTLFEDREMIEERLKTYAHLFDDKVMKQLKRRRYTGWGRL<br>SRKLINGIRDKQSGKTILDFLKSDGFANRNFMQLIHDDSLTFKEDI<br>QKAQVSGQGDSLHEHIANLAGSPAIKKGILQTVKVVDELVKVMG<br>RHKPENIVIEMARENQTTQKGQKNSRERMKRIEEGIKELGSQILK<br>EHPVENTQLQNEKLYLYYLQNGRDMYVDQELDINRLSDYDVDHI<br>VPQSFLKDDSIDNKVLTRSDKNRGKSDNVPSEEVVKKMKNYWR<br>QLLNAKLITQRKFDNLTKAERGGLSELDKAGFIKRQLVETRQITK<br>HVAQILDSRMNTKYDENDKLIREVKVITLKSKLVSDFRKDFQFYK<br>VREINNYHHAHDAYLNAVVGTALIKKYPKLESEFVYGDYKVYDV<br>RKMIAKSEQEIGKATAKYFFYSNIMNFFKTEITLANGEIRKRPLIET<br>NGETGEIVWDKGRDFATVRKVLSMPQVNIVKKTEVQTGGFSKE<br>SILPKRNSDKLIARKKDWDPKKYGGFDSPTVAYSVLVVAKVEKG<br>KSKKLKSVKELLGITIMERSSFEKNPIDFLEAKGYKEVKKDLIIKLP<br>KYSLFELENGRKRMLASAGELQKGNELALPSKYVNFLYLASHYE<br>KLKGSPEDNEQKQLFVEQHKHYLDEIIEQISEFSKRVILADANLD<br>KVLSAYNKHRDKPIREQAENIIHLFTLTNLGAPAAFKYFDTTIDRK<br>RYTSTKEVLDATLIHQSITGLYETRIDLSQLGGD

WT *Spy*Cas9-2NLS in bacterial expression vector	MKSSHHHHHHHHHHGSSMKIEEGKLVIWINGDKGYNGLAEVGK<br>KFEKDTGIKVTVEHPDKLEEKFPQVAATGDGPDIIFWAHDRFGG<br>YAQSGLLAEITPDKAFQDKLYPFTWDAVRYNGKLIAYPIAVEALS<br>LIYNKDLLPNPPKTWEEIPALDKELKAKGKSALMFNLQEPYFTWP<br>LIAADGGYAFKYENGKYDIKDVGVDNAGAKAGLTFLVDLIKNKH<br>MNADTDYSIAEAAFNKGETAMTINGPWAWSNIDTSKVNYGVTVL<br>PTFKGQPSKPFVGVLSAGINAASPNKELAKEFLENYLLTDEGLEA<br>VNKDKPLGAVALKSYEEELAKDPRIAATMENAQKGEIMPNIPQM<br>SAFWYAVRTAVINAASGRQTVDEALKDAQTNSSSNNNNNNNNNN<br>NLGIEENLYFQSNAMDKKYSIGLDIGTNSVGWAVITDEYKVPSKK<br>FKVLGNTDRHSIKKNLIGALLFDSGETAEATRLKRTARRRYTRRK<br>NRICYLQEIFSNEMAKVDDSFFHRLEESFLVEEDKKHERHPIFGN<br>IVDEVAYHEKYPTIYHLRKKLVDSTDKADLRLIYLALAHMIKFRGH<br>FLIEGDLNPDNSDVDKLFIQLVQTYNQLFEENPINASGVDAKAILS<br>ARLSKSRRLENLIAQLPGEKKNGLFGNLIALSLGLTPNFKSNFDL<br>AEDAKLQLSKDTYDDDLDNLLAQIGDQYADLFLAAKNLSDAILLS<br>DILRVNTEITKAPLSASMIKRYDEHHQDLTLLKALVRQQLPEKYK<br>EIFFDQSKNGYAGYIDGGASQEEFYKFIKPILEKMDGTEELLVKL<br>NREDLLRKQRTFDNGSIPHQIHLGELHAILRRQEDFYPFLKDNRE<br>KIEKILTFRIPYYVGPLARGNSRFAWMTRKSEETITPWNFEEVVD<br>KGASAQSFIERMTNFDKNLPNEKVLPKHSLLYEYFTVYNELTKV<br>KYVTEGMRKPAFLSGEQKKAIVDLLFKTNRKVTVKQLKEDYFKKI<br>ECFDSVEISGVEDRFNASLGTYHDLLKIIKDKDFLDNEENEDILED<br>IVLTLTLFEDREMIEERLKTYAHLFDDKVMKQLKRRRYTGWGRL<br>SRKLINGIRDKQSGKTILDFLKSDGFANRNFMQLIHDDSLTFKEDI<br>QKAQVSGQGDSLHEHIANLAGSPAIKKGILQTVKVVDELVKVMG<br>RHKPENIVIEMARENQTTQKGQKNSRERMKRIEEGIKELGSQILK<br>EHPVENTQLQNEKLYLYYLQNGRDMYVDQELDINRLSDYDVDHI<br>VPQSFLKDDSIDNKVLTRSDKNRGKSDNVPSEEVVKKMKNYWR<br>QLLNAKLITQRKFDNLTKAERGGLSELDKAGFIKRQLVETRQITK<br>HVAQILDSRMNTKYDENDKLIREVKVITLKSKLVSDFRKDFQFYK<br>VREINNYHHAHDAYLNAVVGTALIKKYPKLESEFVYGDYKVYDV<br>RKMIAKSEQEIGKATAKYFFYSNIMNFFKTEITLANGEIRKRPLIET<br>NGETGEIVWDKGRDFATVRKVLSMPQVNIVKKTEVQTGGFSKE<br>SILPKRNSDKLIARKKDWDPKKYGGFDSPTVAYSVLVVAKVEKG<br>KSKKLKSVKELLGITIMERSSFEKNPIDFLEAKGYKEVKKDLIIKLP<br>KYSLFELENGRKRMLASAGELQKGNELALPSKYVNFLYLASHYE<br>KLKGSPEDNEQKQLFVEQHKHYLDEIIEQISEFSKRVILADANLD<br>KVLSAYNKHRDKPIREQAENIIHLFTLTNLGAPAAFKYFDTTIDRK<br>RYTSTKEVLDATLIHQSITGLYETRIDLSQLGGDGSPKKKRKVED<br>PKKKRKVDTG

3xFLAG tag; NLS sequences; 10 His-tags; MBP; TEV site; Cas9 protein

Table S7. Protein mutations of Cas9 variants (related to STAR Methods).

Cas9 variants	Protein mutations
WT *Spy*Cas9	
SpG	D1135L/S1136W/G1218K/E1219Q/R1335Q/T1337R
SpRY	A61R/L1111R/D1135L/S1136W/G1218K/E1219Q/N1317R/A1322R/R1333P/R1335Q/T1337R
EQR	D1135E/R1335Q/T1337R
VRER	D1135V/G1218R/R1335E/T1337R
xPBA	R1333A/R1335A
dCas9	D10A/H840A
dSpRY	D10A/A61R/H840A/L1111R/D1135L/S1136W/G1218K/E1219Q/N1317R/A1322R/R1333P/R1335Q/T1337R

Table S8. Example of DNA template sequences used for in-vitro transcription (related to STAR Methods).

	**Sequences (5′-3′)**
sgRNA1 DNA template	TAATACGACTCACTATA**GAGTCCGAGCAGAAGAAGAA**GTTTTAGAGCTAGAAATAGCAAGTTAAAATAAGGCTAGTCCGTTATCAACTTGAAAAAGTGGCACCGAGTCGGTGCTTCG

Underlined region represents the T7 promoter sequence; **bolded region** represents the 20-bp sgRNA spacer; yellow highlighted region represents the sgRNA scaffold sequence.

Table S9. Sequences of all the guide RNA used in this study (related to STAR Methods).

**sgRNA name**	**Internal ID**	**Sequences (5′-3′)**	**Figures**
sgRNA1	rHS_77	**GAGUCCGAGCAGAAGAAGAA**GUUUUAGAGCUAGAAAUAGCAAGUUAAAAUAAGGCUAGUCCGUUAUCAACUUGAAAAAGUGGCACCGAGUCGGUGCUUCG	Figure S2C, S2H, Figure S5E, Figure S5F, Figure S6D
sgRNA1^{AltR}	rHS_76	**mG*mA*mG*UCCGAGCAGAAGAAGAA**GUUUUAGAGCUAGAAAUAGCAAGUUAAAAUAA	Figure 1D, Figure S1E

		GGCUAGUCCGUUAUCAACUUGAAAAAGUGGCACCGAGUCGG*mU*mG*mC	
sgRNA2	rHS_6	GG**GACGCAUAAAGAUGAGACAA**GUUUUAGAGCUAGAAAUAGCAAGUUAAAAUAAGGCUAGUCCGUUAUCAACUUGAAAAAGUGGCACCGAGUCGGUGCUUCG	Figure 1E, F, Figure 3E, Figure 5B-F, Figure 6A, B, Figure S1G-I, Figure S2A, D, Figure S3I, Figure S5A-E, G, Figure S6A-C, Figure S7
sgRNA2.1	rHS_55	GG**GACGCAUAAAGAUGAGAGUU**GUUUUAGAGCUAGAAAUAGCAAGUUAAAAUAAGGCUAGUCCGUUAUCAACUUGAAAAAGUGGCACCGAGUCGGUGCUUCG	Figure S4A, B, E
sgRNA3	rHS_1	GG**CUGCGUAUUUCUACUCUGUU**GUUUUAGAGCUAGAAAUAGCAAGUUAAAAUAAGGCUAGUCCGUUAUCAACUUGAAAAAGUGGCACCGAGUCGGUGCUUCG	Figure 2, Figure 3A-C, Figure 4G-4I, Figure S2E, Figure S3B-H, Figure S5E
sgRNA4	rHS_2	GG**CACACACACACACACACAGG**GUUUUAGAGCUAGAAAUAGCAAGUUAAAAUAAGGCUAGUCCGUUAUCAACUUGAAAAAGUGGCACCGAGUCGGUGCUUCG	Figure S2F
sgRNA4.1	rHS_5	GG**CACACACACACACACACCAA**GUUUUAGAGCUAGAAAUAGCAAGUUAAAAUAAGGCUAGUCCGUUAUCAACUUGAAAAAGUGGCACCGAGUCGGUGCUUCG	Figure 4A-F, Figure S4E-G
sgRNA5	rHS_70	**GACGCAUAAAGAUGAGACGC**GUUUUAGAGCUAGAAAUAGCAAGUUAAAAUAAGGCUAGUCCGUUAUCAACUUGAAAAAGUGGCACCGAGUCGGUGCUUCG	Figure 3E, Figure S2G, Figure S3I
sgRNA6	rHS_83	**GAGUGCUAAGGGAACGUUCA**GUUUUAGAGCUAGAAAUAGCAAGUUAAAAUAAGGCUAGUCCGUUAUCAACUUGAAAAAGUGGCACCGAGUCGGUGCUUCG	Figure 3E, Figure S2I, Figure S3I, Figure S5F
sgRNA6^{AltR}	rHS_81	**mG*mA*mG*UGCUAAGGGAACGUUCA**GUUUUAGAGCUAGAAAUAGCAAGUUAAAAUAAGGCUAGUCCGUUAUCAACUUGAAAAAGUGGCACCGAGUCGG*mU*mG*mC	Figure S1E
sgRNA7^{AltR}	rHS_82	**mG*mG*mU*GCUAGCCUUGCGUUCCG**GUUUUAGAGCUAGAAAUAGCAAGUUAAAAUAAGGCUAGUCCGUUAUCAACUUGAAAAAGUG	Figure S1E

		GCACCGAGUCGG*mU*mG*mC	
sgRNA8	rHS_95	**GUGAUAAGUGGAAUGCCAUG**GUUUUAGAGCUAGAAAUAGCAAGUUAAAAUAAGGCUAGUCCGUUAUCAACUUGAAAAAGUGGCACCGAGUCGGUGCUUCG	Figure S2B

AltR represents chemical modifications of IDT guide RNA; m represents 2'-O methylation; * represents phosphorothioate linkage; **bolded region** represents the 20-bp sgRNA spacer; yellow highlighted region represents the sgRNA scaffold sequence.

Table S10. Sequences of the PCR1 primers for NGS analysis (related to STAR Methods).

**Oligonucleotide**	**Sequence (5′-3′)**
EMX1 Target Forward Primer	**ACACTCTTTCCCTACACGACGCTCTTCCGATCT**TTTCTCATCTGTGCCCCTCCC
EMX1 Target Reverse Primer	**GTGACTGGAGTTCAGACGTGTGCTCTTCCGATCT**GCAGCAAGCAGCACTCTGCC
DNMT1 Target Forward Primer	**ACACTCTTTCCCTACACGACGCTCTTCCGATCT**GGAACACGCCCGGTGTCAC
DNMT1 Target Reverse Primer	**GTGACTGGAGTTCAGACGTGTGCTCTTCCGATCT**CTGGGGCCGTTTCCCTCAC
PCSK9 Target Forward Primer	**ACACTCTTTCCCTACACGACGCTCTTCCGATCT**TCAGCTCCAGGCGGTCCTG
PCSK9 Target Reverse Primer	**GTGACTGGAGTTCAGACGTGTGCTCTTCCGATCT**GGCCCGAGAGGAAACAGCAC

Bolded region represents the Illumina adapter sequences.

Table S11. Sequences of oligonucleotides used for ddPCR (related to STAR Methods).

**Oligonucleotide**	**Sequence (5′-3′)**
EMX1 Target Forward Primer	CCAGAACCGGAGGACAAAGTAC
EMX1 Target Reverse Primer	CCACCCTAGTCATTGGAGGTGAC
EMX1 Target Probe (FAM) Primer	**/56-FAM/**CTGCTTCGT**/ZEN/**GGCAATGCGCCA

	CC**/3IABkFQ/**
EMX1 Reference Forward Primer	GACCACTTGGCCTTCTCCTC
EMX1 Reference Reverse Primer	CACTAAACTACAGTGGTGCCTGG
EMX1 Reference Probe (HEX) Primer	/5HEX/CCGCCCGCC**/ZEN/**ACCGCAGCCTC **/3IABkFQ/**

/56-FAM/ represents 5′-FAM labeling; **/5HEX/** represents 5'-HEX labeling; **/ZEN/** represents internal ZEN quencher; **/3IABkFQ/** represents 3' Iowa Black FQ quencher.

Table S12. Sequences of the primers used for RT-qPCR (related to STAR Methods).

**Oligonucleotide**	**Sequence (5′-3′)**
EMX1 sgRNA Forward Primer	GAGTCCGAGCAGAAGAAGAAG
EMX1 sgRNA Reverse Primer	CTCGGTGCCACTTTTTCAAG
ACTB (β-actin) Forward Primer	ACCTTCTACAATGAGCTGCG
ACTB (β-actin) Reverse Primer	CCTGGATAGCAACGTACATGG

Table S13. Sequences of all the DNA substrates used in biochemical assays (related to STAR Methods).

Internal ID	Description	Sequences (5′-3′)	Figures
HS_1313	DNA substrate 0-20 bp match to sgRNA1; NTS; Cy5	/5Cy5/GGAAGGGCCT**GAGTCCGAGCAGAAGAAGAA**GGGCTCCCATCACATCAACCG	Figure S2C, S2H, Figure S5E-F, Figure S6D
HS_1339	DNA substrate 3-20 bp match to sgRNA1 (3-bp mismatch bubble); NTS; Cy5	/5Cy5/GGAAGGGCCT**GAGTCCGAGCAGAAGAACTT**GGGCTCCCATCACATCAACCG	Figure S6D
HS_1276	DNA substrate 0-20 bp match to sgRNA1; TS; FAM	/56-FAMN/CGGTTGATGTGATGGGAGCCC**TTCTTCTTCTGCTCGGACTC**AGGCCCTTCC	Figure S2C, S2H, Figure S5E-F, Figure S6D
HS_33	DNA substrate 0-20 bp match to sgRNA2; NTS; unlabeled	CGCTCATGCT**GACGCATAAAGATGAGACAA**TGGCGATTACAGTACGTGCG	Figure 4G-I
HS_1198	DNA substrate 0-20 bp match to sgRNA2; NTS; 2AP at position 15 from PAM	CGCTCATGCT**GACGC/i2AmPr/TAAAGATGAGACAA**TGGCGATTACAGTACGTGCG	Figure 1F, Figure S2B
HS_284	DNA substrate 0-20 bp match to sgRNA2; NTS; Cy5	/5Cy5/CGCTCATGCT**GACGCATAAAGATGAGACAA**TGGCGATTACAGTACGTGCG	Figure 1E, Figure 3E, Figure 5B-F, Figure 6A, B, Figure S1G-I, Figure S2A, S2D, Figure S5A-E, G, Figure S6C
HS_500	DNA substrate 3-20 bp match to sgRNA2 (3-bp mismatch bubble); NTS; Cy5	/5Cy5/CGCTCATGCT**GACGCATAAAGATGAGAGTT**TGGCGATTACAGTACGTGCG	Figure 3E, Figure S3I
HS_34	DNA substrate 0-20 bp match to sgRNA2; TS; unlabeled	CGCACGTACTGTAATCGCCA**TTGTCTCATCTTTATGCGTC**AGCATGAGCG	Figure 1F, Figure S2B
HS_285	DNA substrate 0-20 bp match to sgRNA2; TS; FAM	/56-FAMN/CGCACGTACTGTAATCGCCA**TTGTCTCATCTTTATGCGTC**AGCATGAGCG	Figure 1E, Figure 3E, Figure 5B-F, Figure 6A, B, Figure S1G-I,

			Figure S2A, S2D, Figure S3I, Figure S5A-E, G, Figure S6C
HS_459	DNA substrate 0-20 bp match to sgRNA2; NTS; Cy5; NGAG PAM	/5Cy5/CGCTCATGCT**GACGCATAAAGATGAGACAA**TGAGGATTACAGTACGTGCG	Figure S7
HS_360	DNA substrate 0-20 bp match to sgRNA2; TS; FAM; NGAG PAM	/56-FAMN/CGCACGTACTGTAATCCTCA**TTGTCTCATCTTTATGCGTC**AGCATGAGCG	Figure S7
HS_461	DNA substrate 0-20 bp match to sgRNA2; NTS; Cy5; NGCG PAM	/5Cy5/CGCTCATGCT**GACGCATAAAGATGAGACAA**TGCGGATTACAGTACGTGCG	Figure S7
HS_362	DNA substrate 0-20 bp match to sgRNA2; TS; FAM; NGCG PAM	/56-FAMN/CGCACGTACTGTAATCCGCA**TTGTCTCATCTTTATGCGTC**AGCATGAGCG	Figure S7
HS_37	DNA substrate 0-20 bp match to sgRNA3; NTS; Cy5	/5Cy5/CGCTCATGCT**CTGCGTATTTCTACTCTGTT**TGGCGATTACAGTACGTGCG	Figure S2E, Figure S5E
HS_38	DNA substrate 0-20 bp match to sgRNA3; TS; FAM	/56-FAMN/CGCACGTACTGTAATCGCCA**AACAGAGTAGAAATACGCAG**AGCATGAGCG	Figure S2E, Figure S5E
HS_451	DNA substrate 0-20 bp match to sgRNA4; NTS; Cy5	/5Cy5/CGCTCATGCT**CACACACACACACACACAGG**TGGCGATTACAGTACGTGCG	Figure S2F
HS_452	DNA substrate 0-20 bp match to sgRNA4; TS; FAM	/56-FAMN/CGCACGTACTGTAATCGCCA**CCTGTGTGTGTGTGTGTGTG**AGCATGAGCG	Figure S2F
HS_1237	DNA substrate 0-20 bp match to sgRNA5; NTS; Cy5	/5Cy5/CGCTCATGCT**GACGCATAAAGATGAGACGC**TGGCGATTACAGTACGTGCG	Figure 3E, Figure S2G
HS_1338	DNA substrate 3-20 bp match to sgRNA5 (3-bp mismatch bubble); NTS; Cy5	/5Cy5/CGCTCATGCT**GACGCATAAAGATGAGAGCG**TGGCGATTACAGTACGTGCG	Figure 3E, Figure S3I
HS_1238	DNA substrate 0-20 bp match to sgRNA5; TS;	/56-FAMN/CGCACGTACTGTAATCGCCA**GCGTCTCATC**	Figure 3E, Figure S2G,

	FAM	**TTTATGCGTC**AGCATGAGCG	Figure S3I
HS_1316	DNA substrate 0-20 bp match to sgRNA6; NTS; Cy5	/5Cy5/ATAAGTGGCA**GAGTGCTAAGGGAACGTTCA** CGGAGACTGAACACTCCTCA	Figure 3E, Figure S2I, Figure S5F
HS_1342	DNA substrate 3-20 bp match to sgRNA6 (3-bp mismatch bubble); NTS; Cy5	/5Cy5/ATAAGTGGCA**GAGTGCTAAGGGAACGTAGT** CGGAGACTGAACACTCCTCA	Figure 3E, Figure S3I
HS_1278	DNA substrate 0-20 bp match to sgRNA6; TS; FAM	/56-FAMN/TGAGGAGTGTTCAGTCTCCG**TGAACGTTCC** **CTTAGCACTC**TGCCACTTAT	Figure 3E, Figure S2I, Figure S3I, Figure S5F,
HS_721	DNA competitor contains 10 NGG; strand 1	CAATGGCAATGGCAATGGCAATGGCAATGGCAAT GGCAATGGCAATGGCAATGGCAATGG	Figure 5F, Figure S5G
HS_722	DNA competitor contains 10 NGG; strand 2	CCATTGCCATTGCCATTGCCATTGCCATTGCCATT GCCATTGCCATTGCCATTGCCATTG	Figure 5F, Figure S5G
HS_723	DNA competitor contains 5 NGG; strand 1	CAATGGCAATTTCAATGGCAATTTCAATGGCAATTT CAATGGCAATTTCAATGGCAATTT	Figure 5F, Figure S5G
HS_724	DNA competitor contains 5 NGG; strand 2	AAATTGCCATTGAAATTGCCATTGAAATTGCCATTG AAATTGCCATTGAAATTGCCATTG	Figure 5F, Figure S5G
HS_775	DNA competitor contains 2 NGG; strand 1	CAATTTCAATTTCAATGGCAATTTCAATTTCAATTT CAATGGCAATTTCAATTTCAATTT	Figure 5F, Figure S5G
HS_776	DNA competitor contains 2 NGG; strand 2	AAATTGAAATTGAAATTGCCATTGAAATTGAAATTG AAATTGCCATTGAAATTGAAATTG	Figure 5F, Figure S5G
HS_727	DNA competitor contains 0 NGG; strand 1	CAATTTCAATTTCAATTTCAATTTCAATTTCAATTTC AATTTCAATTTCAATTTCAATTT	Figure 5F, Figure S5G
HS_728	DNA competitor contains 0 NGG; strand 2	AAATTGAAATTGAAATTGAAATTGAAATTGAAATTG AAATTGAAATTGAAATTGAAATTG	Figure 5F, Figure S5G

/56-FAMN/ represents 5′-FAM labeling; /5Cy5/ represents 5′-Cy5 labeling; /i2AmPr/ represents internal 2AP labeling; **bolded region** represents the 20-bp sgRNA spacer; yellow highlighted region represents the NGG PAM for WT *Spy*Cas9, NGAG for EQR, NGCG for VRER

Table S14. Sequences of the plasmid competitor (related to STAR Methods).

	**Sequences (5′-3′)**
pGGAselect	CGAAAAATCAATAATCAGACAACAAGATGTGCGAACTCGATATTTT<br>ACACGACTCTCTTTACCAATTCTGCCCCGAATTACACTTAAAACGA<br>CTCAACAGCTTAACGTTGGCTTGCCACGCATTACTTGACTGTAAAA<br>CTCTCACTCTTACCGAACTTGGCCGTAACCTGCCAACCAAAGCGA<br>GAACAAAACATAACATCAAACGAATCGACCGATTGTTAGGTAATCG<br>TCACCTGCAGGAAGGTTTAAACGCATTTAGGTGACACTATAGAAGT<br>GTGTATCGCTCGAGGGATCCGAATTCGAAGTCTTGGTACGGAGCG<br>AGACCGGAGCGAGACGGGAGTCGTCTTCGCTTTCCAGATCTGATA<br>ACTTGTGAAGACGACCATCGTCTCACCATGGTCTCACCATTCCTGT<br>AGACTTCTTAATTAAGACGTCAGAATTCTCGAGGCGGCCGCATGT<br>GAGTCTCCCTATAGTGAGTCGTATTAATTTCGCGGGCGGAACCCC<br>TATTTGTTTATTTTTCTAAATACATTCAAATATGTATCCGCTCATGAG<br>TAGCACCAGGCGTTTAAGGGCACCAATAACTGCCTTAAAAAAATTA<br>CGCCCCGCCCTGCCACTCATCGCAGTACTGTTGTAATTCATTAAG<br>CATTCTGCCGACATGGAAGCCATCACAAACGGCATGATGAACCTG<br>AATCGCCAGCGGCATCAGCACCTTGTCGCCTTGCGTATAATATTTG<br>CCCATGGTGAAAACGGGGGCGAAGAAGTTGTCCATATTGGCCACG<br>TTTAAATCAAAACTGGTGAAACTCACCCAGGGATTGGCTGAGACAA<br>AAAACATATTCTCAATAAACCCTTTAGGGAAATAGGCCAGGTTTTC<br>ACCGTAACACGCCACATCTTGCGAATATATGTGTAGAAACTGCCG<br>GAAATCGTCGTGGTATTCACTCCAGAGCGATGAAAACGTTTCAGTT<br>TGCTCATGGAAAACGGTGTAACAAGGGTGAACACTATCCCATATCA<br>CCAGCTCACCGTCTTTCATTGCCATACGAAATTCCGGATGAGCATT<br>CATCAGGCGGGCAAGAATGTGAATAAAGGCCGGATAAAACTTGTG<br>CTTATTTTTCTTTACGGTCTTTAAAAAGGCCGTAATATCCAGCTGAA<br>CGGTCTGGTTATAGGTACATTGAGCAACTGACTGAAATGCCTCAAA<br>ATGTTCTTTACGATGCCATTGGGATATATCAACGGTGGTATATCCA<br>GTGATTTTTTTCTCCATTTTAGCTTCCTTAGCTCCTGAAAATCTCGA<br>TAACTCAAAAAATACGCCCGGTAGTGATCTTATTTCATTATGGTGA<br>AAGTTGGAACCTCTTACGTGCCGATCAAAGTCTCATTTTCGCCAAA<br>AGTTGTCATGACCAAAATCCCTTAACGTGAGTTTTCGTTCCACTGA<br>GCGTCAGACCCCGTAGAAAAGATCAAAGGATCTTCTTGAGATCCTT<br>TTTTTCTGCGCGTAATCTGCTGCTTGCAAACAAAAAAACCACCGCT<br>ACCAGCGGTGGTTTGTTTGCCGGATCAAGAGCTACCAACTCTTTTT<br>CCGAAGGTAACTGGCTTCAGCAGAGCGCAGATACCAAATACTGTT<br>CTTCTAGTGTAGCCGTAGTTAGGCCACCACTTCAAGAACTCTGTAG<br>CACCGCCTACATACCTCGCTCTGCTAATCCTGTTACCAGTGGCTG<br>CTGCCAGTGGCGATAAGTCGTGTCTTACCGGGTTGGACTCAAGAC

	GATAGTTACCGGATAAGGCGCAGCGGTCGGGCTGAACGGGGGGT TCGTGCACACAGCCCAGCTTGGAGCGAACGACCTACACCGAACTG AGATACCTACAGCGTGAGCTATGAGAAAGCGCCACGCTTCCCGAA GGGAGAAAGGCGGACAGGTATCCGGTAAGCGGCAGGGTCGGAAC AGGAGAGCGCACGAGGGAGCTTCCAGGGGGAAACGCCTGGTATC TTTATAGTCCTGTCGGGTTTCGCCACCTCTGACTTGAGCGTCGATT TTTGTGATGCTCGTCAGGGGGGCGGAGCCTATGGAAAAACGCCA GCAATGCGGCCTTTTTACGGTTCCTGGCCTTTTGCTGGCCTTTTGC TCACATGTTCTTTCCTGCGTTATCCCCTGATTCTGTGGATAACCGT ATTACCGCCTTTGAGTGAGCTGATACCGCTCGCCGCAGCCGAACG ACCGAGCGCAGCGAGTCAGTGAGCGAGGAAGC

Table S15. PCR primers and templates used for DNA tether construction for AuRBT experiments (related to STAR Methods).

Name	Primer sequence (5′-3′)	Length (bps)	Template	Digest
XD	FW primer:<br>gaagggtctcaTGACTCACTA**T**AGGGCGAAT**T**GGAGCTCCACCGCG<br><br>RW primer:<br>/5FluorT/CACTAAAGGGAACAAAAGCTGGTAC	4140	pFO-SE2	BsaI
SPD200	FW primer:<br>gaagggtctcaCACTATTAAGTCGAAACAGTCGAGCGTGAAC<br><br>RW primer:<br>gaagggtctcaGCTCGCGACGTTGCGCTACGTCAT	212	pII-lam1	BsaI
SPMDIG	FW primer:<br>GCGCAGCACGCAGATAAATTC<br><br>RW primer:<br>gatcggtctccAGTGCAAACGTCTGCGTCGCTG	308	pII-lam1	BsaI

Internal biotin-modified nucleotides (/iBiodT/) are colored red; /5FluorT/ represents 5'-Fluorescein dT.

Table S16. DNA oligonucleotides used to incorporate sequences of interest (SOI) into DNA tethers for AuRBT experiments (related to STAR Methods).

Name	Target strand (5′-3′)	Nontarget strand (5′-3′)	Length (bps)
Target1: NGG	/5Phos/gtcaTTGTCGCGAAGT<br>GCAGCGAGATCGCGTTTGT<br>ACTCCA**AACAGAGTAGAAA**<br>**TACGCAG**AGCAGCGATTTC<br>TCGTGTGCAGACA	/5Phos/gagcTGTCTGCACACG<br>AGAAATCGCTGCT**CTGCGTA**<br>**TTTCTACTCTGTT**TGGAGTAC<br>AAACGCGATCTCGCTGCACT<br>TCGCGACAA	86
Target1: NCG	/5Phos/gtcaTTGTCGCGAAGT<br>GCAGCGAGATCGCGTTTGT<br>ACTCGA**AACAGAGTAGAAA**<br>**TACGCAG**AGCAGCGATTTC<br>TCGTGTGCAGACA	/5Phos/gagcTGTCTGCACACG<br>AGAAATCGCTGCT**CTGCGTA**<br>**TTTCTACTCTGTT**TCGAGTAC<br>AAACGCGATCTCGCTGCACT<br>TCGCGACAA	86
Target2: NGG	/5Phos/gtcaTTGTCGCGAAGT<br>GCAGCGAGATCGCGTTTGT<br>ACTCCA**TTGTCTCATCTTTA**<br>**TGCGTC**AGCAGCGATTTCT<br>CGTGTGCAGACA	/5Phos/gagcTGTCTGCACACG<br>AGAAATCGCTGCT**GACGCAT**<br>**AAAGATGAGACAA**TGGAGT<br>ACAAACGCGATCTCGCTGCA<br>CTTCGCGACAA	86
Target2: NCG	/5Phos/gtcaTTGTCGCGAAGT<br>GCAGCGAGATCGCGTTTGT<br>ACTCGA**TTGTCTCATCTTTA**<br>**TGCGTC**AGCAGCGATTTCT<br>CGTGTGCAGACA	/5Phos/gagcTGTCTGCACACG<br>AGAAATCGCTGCT**GACGCAT**<br>**AAAGATGAGACAA**TCGAGT<br>ACAAACGCGATCTCGCTGCA<br>CTTCGCGACAA	86

/5Phos/ represents 5’-phosphorylation; **bolded region** represents the 20-bp sgRNA spacer;

yellow highlighted region represents the PAM.

Table S17. Example DNA tether used for AuRBT experiments (related to STAR Methods).

	Target strand (5’-3’)
Target2: NGG	/5FluorT/CACTAAAGGGAACAAAAGCTGGTACCGGGCCCCCCCTCGAGCGGT<br>ACCCCACTTACCCACCCCGGAAATTTGAGTTATAAACGTTGTTTGAGCTTTAC<br>CTAGTCTTGGTCGATCAAAAGTTCTGGTACCTTTTCACCATGTCTCCCCCCTT<br>ATTCATATAAAAAGAAGCGTATAATCGCACAGTATAACGCTCCTCTGATATAT<br>GATCTAGACCCAAGTAATGAGTTACGAATCTGGGAGGTCATCCTCCTCTTCC<br>GAGAGTACACGGCCACCAACGCTAAAAGAAGAACCTAATGGTAAAATAGCTT<br>GGGAAGAAAGTGTCAAAAAATCTAGGGAAAATAACGAAAATGACAGCACTCT<br>CTTGAGGCGAAAGCTAGGTGAGACTCGAAAAGCAATTGAAACTGGAGGATC<br>ATCGAGAAATAAACTTTCTGCTTTGACACCCTTGAAAAAAGTGGTTGACGAGA<br>GGAAGGATTCGGTACAACCACAGGTCCCTTCCATGGGTTTTACTTATTCTTT<br>GCCTAATTTGAAGACTTTAAACAGTTTTTCAGATGCTGAGCAAGCACGTATAA<br>TGCAAGATTATCTATCCAGGGGGGTAAATCAAGGCAACAGTAATAATTATGTA<br>GACCCACTATATCGGCAATTAAATCCAACTATGGGTAGTAGCAGGAACAGGC

	CTGTTTGGAGTTTAAATCAGCCGTTACCGCATGTATTGGATCGAGGCTTGGC<br>AGCAAAGATGATACAAAAGAATATGGATGCAAGGTCCCGCGCATCATCGAGA<br>CGAGGGTCGACCGATATTTCAAGGGGGGGTTCTACTACGTCAGTGAAAGAC<br>TGGAAAAGGCTCCTTAGAGGTGCAGCACCGGGTAAAAAGCTTGGTGACATC<br>GAAGCTCAAACGCAACGCGATAATACTGTTGGTGCAGATGTGAAACCTACTA<br>AGTTAGAGCCTGAAAACCCACAAAAGCCCTCTAACACGCATATTGAGAATGT<br>TTCACGTAAGAAAAAGCGTACTTCGCATAATGTCAATTTTTCATTAGGCGATG<br>AAAGCTACGCATCCTCCATAGCCGATGCAGAATCCAGAAAATTAAAGAACAT<br>GCAAACCCTCGATGGTTCTACTCCGGTTTATACGAAGCTTCCTGAAGAACTT<br>ATTGAAGAGGAAAATAAAAGTACGAGTGCATTAGATGGTAATGAAATTGGTG<br>CCTCAGAAGATGAAGACGCGGATATAATGACATTTCCTAACTTTTGGGCAAA<br>AATTCGCTATCATATGCGAGAACCGTTTGCGGAGTTTCTCGGGACACTAGTT<br>CTTGTCATTTTTGGTGTTGGTGGTAATCTTCAAGCAACTGTAACAAAAGGTAG<br>TGGTGGTTCCTATGAATCCCTATCATTTGCATGGGGGTTCGGTTGTATGCTT<br>GGTGTTTACGTCGCAGGCGGTATTAGTGGTGGTCATATTAACCCTGCTGTTA<br>CGATTTCAATGGCAATTTTTCGAAAATTCCCCTGGAAAAAGGTGCCCGTATAT<br>ATTGTTGCTCAGATTATCGGTGCATATTTTGGAGGAGCTATGGCTTATGGTTA<br>TTTTTGGAGCTCTATCACAGAATTTGAGGGAGGTCCGCACATAAGAACAACG<br>GCGACCGGTGCGTGTTTGTTTACTGATCCAAAGTCTTACGTCACGTGGAGAA<br>ATGCCTTCTTTGACGAATTCATAGGAGCCTCTATACTTGTGGGTTGTTTGATG<br>GCGCTATTGGATGATAGTAATGCTCCACCTGGCAATGGTATGACCGCATTAA<br>TTATTGGATTCTTAGTCGCTGCAATTGGTATGGCCCTTGGATATCAAACAAGT<br>TTCACAATCAATCCTGCAAGAGATCTCGGTCCTCGCATATTTGCTTCCATGAT<br>TGGCTATGGTCCACATGCTTTTCATCTCACACATTGGTGGTGGACATGGGGA<br>GCCTGGGGTGGTCCAATTGCCGGCGGTATTGCTGGAGCACTCATATATGAC<br>ATTTTCATTTTTACTGGATGCGAATCCCCAGTCAACTACCCAGACAACGGTTA<br>TATTGAGAATAGGGTAGGCTGCAGCCCGGGGGATCCACTAGTTCTAGAGCG<br>GCCGCCGGTACCCCACTTACCCACCCCGGAAATTTGAGTTATAAACGTTGTT<br>TGAGCTTTACCTAGTCTTGGTCGATCAAAAGTTCTGGTACCTTTTCACCATGT<br>CTCCCCCCTTATTCATATAAAAAGAAGCGTATAATCGCACAGTATAACGCTCC<br>TCTGATATATGATCTAGACCCAAGTAATGAGTTACGAATCTGGGAGGTCATC<br>CTCCTCTTCCGAGAGTACACGGCCACCAACGCTAAAAGAAGAACCTAATGGT<br>AAAATAGCTTGGGAAGAAAGTGTCAAAAAATCTAGGGAAAATAACGAAAATG<br>ACAGCACTCTCTTGAGGCGAAAGCTAGGTGAGACTCGAAAAGCAATTGAAAC<br>TGGAGGATCATCGAGAAATAAACTTTCTGCTTTGACACCCTTGAAAAAAGTG<br>GTTGACGAGAGGAAGGATTCGGTACAACCACAGGTCCCTTCCATGGGTTTTA<br>CTTATTCTTTGCCTAATTTGAAGACTTTAAACAGTTTTTCAGATGCTGAGCAA<br>GCACGTATAATGCAAGATTATCTATCCAGGGGGGTAAATCAAGGCAACAGTA<br>ATAATTATGTAGACCCACTATATCGGCAATTAAATCCAACTATGGGTAGTAGC<br>AGGAACAGGCCTGTTTGGAGTTTAAATCAGCCGTTACCGCATGTATTGGATC<br>GAGGCTTGGCAGCAAAGATGATACAAAAGAATATGGATGCAAGGTCCCGCG<br>CATCATCGAGACGAGGGTCGACCGATATTTCAAGGGGGGGTTCTACTACGT<br>CAGTGAAAGACTGGAAAAGGCTCCTTAGAGGTGCAGCACCGGGTAAAAAGC<br>TTGGTGACATCGAAGCTCAAACGCAACGCGATAATACTGTTGGTGCAGATGT<br>GAAACCTACTAAGTTAGAGCCTGAAAACCCACAAAAGCCCTCTAACACGCAT<br>ATTGAGAATGTTTCACGTAAGAAAAAGCGTACTTCGCATAATGTCAATTTTTC<br>ATTAGGCGATGAAAGCTACGCATCCTCCATAGCCGATGCAGAATCCAGAAAA<br>TTAAAGAACATGCAAACCCTCGATGGTTCTACTCCGGTTTATACGAAGCTTCC<br>TGAAGAACTTATTGAAGAGGAAAATAAAAGTACGAGTGCATTAGATGGTAAT<br>GAAATTGGTGCCTCAGAAGATGAAGACGCGGATATAATGACATTTCCTAACT

	TTTGGGCAAAAATTCGCTATCATATGCGAGAACCGTTTGCGGAGTTTCTCGG<br>GACACTAGTTCTTGTCATTTTTGGTGTTGGTGGTAATCTTCAAGCAACTGTAA<br>CAAAAGGTAGTGGTGGTTCCTATGAATCCCTATCATTTGCATGGGGGTTCGG<br>TTGTATGCTTGGTGTTTACGTCGCAGGCGGTATTAGTGGTGGTCATATTAAC<br>CCTGCTGTTACGATTTCAATGGCAATTTTTCGAAAATTCCCCTGGAAAAAGGT<br>GCCCGTATATATTGTTGCTCAGATTATCGGTGCATATTTTGGAGGAGCTATG<br>GCTTATGGTTATTTTTGGAGCTCTATCACAGAATTTGAGGGAGGTCCGCACA<br>TAAGAACAACGGCGACCGGTGCGTGTTTGTTTACTGATCCAAAGTCTTACGT<br>CACGTGGAGAAATGCCTTCTTTGACGAATTCATAGGAGCCTCTATACTTGTG<br>GGTTGTTTGATGGCGCTATTGGATGATAGTAATGCTCCACCTGGCAATGGTA<br>TGACCGCATTAATTATTGGATTCTTAGTCGCTGCAATTGGTATGGCCCTTGGA<br>TATCAAACAAGTTTCACAATCAATCCTGCAAGAGATCTCGGTCCTCGCATATT<br>TGCTTCCATGATTGGCTATGGTCCACATGCTTTTCATCTCACACATTGGTGGT<br>GGACATGGGGAGCCTGGGGTGGTCCAATTGCCGGCGGTATTGCTGGAGCA<br>CTCATATATGACATTTTCATTTTTACTGGATGCGAATCCCCAGTCAACTACCC<br>AGACAACGGTTATATTGAGAATAGGGTAGGGGCCGCCACCGCGGTGGAGCT<br>CC**A**ATTCGCCCT**A**TAGTGAGTCATTGTCGCGAAGTGCAGCGAGATCGCGTTT<br>GTACTCCA**TTGTCTCATCTTTATGCGTC**AGCAGCGATTTCTCGTGTGCAGAC<br>AGCTCGCGACGTTGCGCTACGTCATCTAGTAGTGCGAACTGACTCTCAGTAT<br>TCGACAAATCTTGACGCTGCTGACTGATTTTCTCATTTGCTCATGCATGCTTC<br>TCTAGCGTCAGTAGTTCTGACTGAAGCGTCAGCAGCTCAGCATGAGCACTGT<br>CTAGATGACGATCGCTCGCAGACACGTTCACGCTCGACTGTTTCGACTTAAT<br>AGTGCAAACGTCTGCGTCGCTGCACTTTTTACGTGCGACATACTGTCAGTCG<br>CGCTCTCTTCGCACTCACTCGAGCTGAACTTCAGCAGTGCGATACAGTTCGC<br>TCGAAGCTGCTCTTCAGCTGCTCAGCTCTTTTTCTGCTCTGACATGACGTTAT<br>TCAGCGCGAGCGTATTATCGCGATACTGTTCGTTCACGCGCTGTTCAGTGCG<br>TTGCAGTTCTGCGTGCAGCTCAGTCAGCACTTCACTTTTCGCATCAATGATT<br>GACTGTTTTGCACGTTGCTGCTGTGCGAATTTATCTGCGTGCTGCGC

/5FluorT/ represents 5’-Fluorescein dT; **A** represents adenine nucleotides paired with biotin-modified thymines, where the streptavidin-coated gold rotor bead could be attached; **bolded region** represents the 20-bp sgRNA spacer; yellow highlighted region represents the PAM; cyan highlighted region represents the segment with multiple incorporated dUTP-digoxigenin (Roche).

Table S18. AuRBT trace statistics (related to Fig. 2D, 3A, Fig. S3E-H, and STAR Methods).

[dCas9 : sgRNA3] (nM)	Tethers	Unique Chambers	Time Tracked (s)	C → I	I → C	I → O	O → I	C → O	O → C
0.8	3	3	17745.75	81 (98.8%)	81 (5%)	1539 (95%)	1537 (99.9%)	1 (1.2%)	1 (0.1%)
4	1	1	1350	21 (100%)	21 (10.6%)	177 (89.4%)	176 (100%)	0 (0%)	0 (0%)

8	1	1	4600	51 (100%)	51 (6.7%)	714 (93.3%)	714 (100%)	0 (0%)	0 (0%)
80	2	1	3333.45	49 (100%)	49 (7.8%)	580 (92.2%)	578 (100%)	0 (0%)	0 (0%)
**[dSpRY : sgRNA3] (nM)**	**Tethers**	**Unique Chambers**	**Time Tracked (s)**	**C → I**	**I → C**	**I → O**	**O → I**	**C → O**	**O → C**
0.8	1	1	1328.91	5 (100%)	6 (15.4%)	33 (84.6%)	33 (100%)	0 (0%)	0 (0%)
1.6	1	1	1214.61	7 (100%)	8 (14.8%)	46 (85.2%)	46 (100%)	0 (0%)	0 (0%)
4	6	6	25636.39	200 (99.5%)	206 (28.2%)	525 (71.8%)	525 (99.8%)	1 (0.5%)	1 (0.2%)
8	2	2	5247.02	55 (98.2%)	54 (25%)	162 (75%)	159 (98.8%)	1 (1.8%)	2 (1.2%)
20	2	2	5712.22	69 (98.6%)	69 (26.2%)	194 (73.8%)	192 (99.5%)	1 (1.4%)	1 (0.5%)
40	2	2	6211.5	88 (98.9%)	88 (15.6%)	476 (84.4%)	475 (99.8%)	1 (1.1%)	1 (0.2%)
100	4	3	8233.13	145 (99.3%)	147 (13.8%)	918 (86.2%)	917 (100%)	1 (0.7%)	0 (0%)
250	1	1	3550	44 (100%)	44 (26.8%)	120 (73.2%)	119 (99.2%)	0 (0%)	1 (0.8%)
500	2	1	3688.25	85 (100%)	86 (40.6%)	126 (59.4%)	125 (100%)	0 (0%)	0 (0%)

The number of independent DNA tethers and flow chambers are tabulated for each condition. In addition, the number of scored transition events are indicated for each type of transition *i* → *j* (with percentages indicating the fraction of transitions starting in state *i* that end in state *j*).

Table S19. AuRBT trace statistics (related to Fig. 4B, 4D, Fig. S4A-C, E and STAR Methods).

**Condition**	**Tethers**	**Unique Chambers**	**Time Tracked (s)**	**C → U**	**U → C**	**C → V**	**V → C**	**V → U**	**U → V**
Target2: NGG / WT : sgRNA4.1	3	3	7428.74	256	259	3	1	1	3
Target2: NCG / WT : sgRNA4.1	3	2	6995.92	1	1	2	2	0	0
Target1: NGG / WT : sgRNA2.1	6	6	18685.14	267	269	18	15	5	3

Target1: NCG / WT : sgRNA2.1	4	3	13229.94	3	3	11	11	0	0
Target1: NGG / 100 nM apo WT	3	2	3104.75	14	14	18	18	1	1
Target1: NGG / 1 uM apo WT	2	2	2816	11	10	11	11	0	0

Here, the transition to “U” indicates unwinding of the DNA beyond 1 bp. To faithfully capture the asymmetry of the raw scored data, we also counted scored states where the DNA was overwound by more than 1 bp (see Methods). Overwound states are denoted by “V”.

Table S20. AuRBT trace statistics (related to Fig. 4F and STAR Methods).

Target2: NGG / [SpRY : sgRNA 4.1] (nM)	Tethers	Unique Chambers	Time Tracked (s)
1	1	1	1107.25
4	4	3	12407.47
5	1	1	1073.51
10	1	1	1079.19
25	1	1	1067.28
50	6	5	9734.46
100	1	1	1087.93
250	1	1	1069.86
**Target2: NCG / [SpRY : sgRNA 4.1] (nM)**	**Tethers**	**Unique Chambers**	**Time Tracked (s)**
2.5	1	1	2501.59
4	3	2	13030.29
10	1	1	4550.91

Supplemental References

1. Cofsky, J.C., Soczek, K.M., Knott, G.J., Nogales, E., and Doudna, J.A. (2022). CRISPR-Cas9 bends and twists DNA to read its sequence. Nature structural & molecular biology *29*, 395-402. 10.1038/s41594-022-00756-0.
2. Zhou, H., Hintze, B.J., Kimsey, I.J., Sathyamoorthy, B., Yang, S., Richardson, J.S., and Al-Hashimi, H.M. (2015). New insights into Hoogsteen base pairs in DNA duplexes from a structure-based survey. Nucleic acids research *43*, 3420-3433. 10.1093/nar/gkv241.
3. Woody, M.S., Lewis, J.H., Greenberg, M.J., Goldman, Y.E., and Ostap, E.M. (2016). MEMLET: An Easy-to-Use Tool for Data Fitting and Model Comparison Using Maximum-Likelihood Estimation. Biophysical journal *111*, 273-282. 10.1016/j.bpj.2016.06.019.
4. Singh, D., Sternberg, S.H., Fei, J., Doudna, J.A., and Ha, T. (2016). Real-time observation of DNA recognition and rejection by the RNA-guided endonuclease Cas9. Nature communications *7*, 12778. 10.1038/ncomms12778.
