# Supplementary material for: Rapid two-step target capture ensures efficient CRISPR-Cas9-guided genome editing
Source: Mol Cell. Author manuscript; Available in PMC 2025 Jul 14. (PMC12258621; doi:10.1016/j.molcel.2025.03.024)
Supplement: 2 [file NIHMS2082181-supplement-2.pdf]

Supplemental Notes

Note 1. A kinetic model of target capture by dCas9 and dSpRY

Our kinetic model (Fig. 3B) integrates experimental findings with prior Cas9 kinetic studies[6,11,20,24,32] to explain differences between dCas9 and dSpRY observed in AuRBT experiments. Analysis of AuRBT yields measurements of four transition rates for each enzyme and [RNP] condition: $k_{C \to I}$, $k_{I \to C}$, $k_{I \to O}$, and $k_{O \to I}$. Three of these transition rates were found to be approximately [RNP]-independent as expected for the model, and these measured rates correspond directly to model parameters: $k_{I \to C}$, $k_{I \to O}$, and $k_{O \to I}$. The remaining measured rate $k_{C \to I}$ varies with [RNP], which was modeled by separating the directly observed C state into the substates C_{free} and C_{bound}. Our measurements of $k_{C \to I}$ versus [RNP] (Fig. 3A) are modeled by considering a two-step process (Fig. 3B):

1. **Initial DNA binding ($C_{free} \leftrightarrow C_{bound}$)**: This step is characterized by the equilibrium dissociation constant $K_{d,init}$ which can be expressed as:

$$K_{d,init} = \frac{k_{off,init}}{k_{on,init}}$$

, where $k_{on,init}$ is the association rate of initial binding, and $k_{off,init}$ is the dissociation rate.

2. **Conformational transition ($C_{bound} \leftrightarrow I$)**: This step involves DNA unwinding to form the *R*-loop seed intermediate. The forward and reverse rates for the transition are k_{open} and k_{close} (k_{close} equal to $k_{I \to C}$), respectively.

Every scored dwell in C must begin in C_{bound} since the enzyme has just returned from I. Therefore, the measured transition rate $k_{C \to I}$ corresponds to the inverse of the waiting time for reaching state I starting in C_{bound}, after possible reversible transitions to C_{free} via dissociation and reassociation. It can be shown that this gives the hyperbolic relationship:

$$k_{C \to I} = \frac{k_{open}}{K_{d,init} + [RNP]} \times [RNP]$$

Journal Pre-proof

Note the similarity of this expression to the Michaelis-Menten-like relationship for the overall rate of reaching I from C_{free} :

$$k_{C_{free}\to I} = \frac{k_{open}}{K_{1/2} + [RNP]} \times [RNP]$$

where $K_{1/2} = \frac{k_{off,init}+k_{open}}{k_{on,init}}$. The two expressions above are equivalent for $k_{off,init}$ >> k_{open}.

For dSpRY, the parameters $K_{d,init}$(dSpRY) and k_{open}(dSpRY) are obtained directly from the fit to the hyperbolic saturation curve. At low [RNP], the modeled relationship between $k_{C\to I}$ and [RNP] is approximately linear with slope $k_{on,eff}(dSpRY) = \frac{k_{open}(dSpRY)}{K_{d,init}(dSpRY)}$ = 0.006 $nM^{-1}s^{-1}$ while at high [RNP], $k_{C\to I}$ plateaus and approaches k_{open}(dSpRY) = 0.06 s^{-1}.

For dCas9, we measure a linear relationship without reaching saturation, and therefore the fit does not separately determine $K_{d,init}$(dCas9) and k_{open}(dCas9) but only the ratio $k_{on,eff}(dCas9) = \frac{k_{open}(dCas9)}{K_{d,init}(dCas9)}$ = 0.05 $nM^{-1}s^{-1}$. Since our measurements remain within the linear regime, we can directly constrain $K_{d,init}$(dCas9) >> 80 nM, the highest [dCas9] sampled. Similarly, we can directly constrain k_{open}(dCas9) >> 4 s^{-1}, the highest $k_{C\to I}$ measured. Based on prior literature[6,20,24,32], we propose a higher lower bound $K_{d,init}$(dCas9) > 1 μM, which implies k_{open}(dCas9) > 50 s^{-1} due our measurement of $\frac{k_{open}}{K_{d,init}}$.

In this approximate treatment, the DNA segment in the vicinity of the target is treated as a site that can be unoccupied (C_{free}), or occupied by an RNP in one of three states (C_{bound}, I, or O). Short-range 1D sliding[13] is not treated explicitly, and the C_{bound} state may be understood as an ensemble of configurations where the enzyme may be in different registries and engagement modes. The free [RNP] is assumed to be equal to the concentration injected into the chamber, given the large excess of RNP over DNA in the single-molecule experiment, and [RNP] is not corrected for specific activity, which does not affect the relative comparison between dSpRY and dCas9 since their measured active fractions are equivalent. Interactions between enzymes

Journal Pre-proof

on the DNA, which could occur at high [RNP] for dSpRY[20] are also not considered in this simple model.

Journal Pre-proof

Note 2. Analysis of ChIP-seq of dCas9 and dSpRY in human cells

ChIP-seq analysis of dCas9 and dSpRY revealed distinct binding profiles. dCas9 showed strong enrichment at the target site (Fig. S6F-G) and higher reads per kilobase per million mapped reads (RPKM) to its specific seed and PAM sequence (5'-AAGAANGG-3') (Fig. S6E-G). This suggests dCas9's high specificity for PAM-mediated binding.

In contrast, dSpRY did not show enrichment at the target site (Fig. S6F-G), instead displaying uniform RPKM across regions with and without its corresponding seed and PAM motifs (5'-AAGAANRN-3') (Fig. S6F-G). This pattern suggests a non-specific binding behavior, likely due to dSpRY's broader PAM recognition. Although the uniform coverage might partly result from background noise or lower RNP levels due to reduced sgRNA expression (Fig. S6F-G), it highlights dSpRY's less specific targeting compared to dCas9.

Journal Pre-proof
